# Restoring sight in choice blindness: pupillometry and behavioral evidence of covert detection

**DOI:** 10.3389/fpsyg.2025.1598254

**Published:** 2025-12-04

**Authors:** Pablo R. Grassi, Lena Hoeppe, Emre Baytimur, Andreas Bartels

**Affiliations:** 1 Department of Psychology, University of Tübingen, Tübingen, Germany; 2 Centre for Integrative Neuroscience, Tübingen, Germany; 3 Max-Planck Institute for Biological Cybernetics, Tübingen, Germany; 4 Graduate Training Centre of Neuroscience, University of Tübingen, Tübingen, Germany; 5 Department of Economics, Zurich Center for Neuroeconomics, University of Zurich, Zurich, Switzerland

**Keywords:** choice blindness, pupillometry, introspection, decision making, self-knowledge, covert detection

## Abstract

Intriguing results from “choice blindness” (CB) experiments have shown that when people make choices, but are presented with a false outcome, many seem not to notice the mismatch and even provide reasons for choices they never made. They appear to be “blind” about their intentions. Yet, this effect goes against decision-making accounts and experience, in which we regularly notice outcomes that do not match our choices (e.g., when ordering food). Here, we ask whether participants really fail to detect the manipulation, or whether CB can be accounted for by *covert detection*, in that participants detect changes, but do not report them. To test this, we measured pupil dilation during the experiments to quantify objective responses in addition to reports by participants. In both experiments, we consistently observed that participants failed to report detected mismatches. Moreover, we observed increased pupil dilation during all manipulated trials, irrespective of whether they were reported or not. Thus, we provide conclusive evidence of covert detection in CB. In addition, we show that CB is strongly modulated by the idiosyncrasies of the experimental design. Our results cast doubt on the general validity of CB, and with that on key conclusions of previous studies. Instead, our results suggest no failure of detection, but instead higher-level, cognitively or socially driven hesitance of reporting. Our evidence leads us to a cautious discussion of CB and provides an account that no longer violates our intuitions about human intentionality and rationality, in that participants are less introspectively blind than originally portrayed.

## Introduction

The ability to monitor and evaluate the outcomes of our decisions and actions is essential for flexible, goal-directed behavior (Botvinick et al., 2004; Walton et al., 2004; Alexander and Brown, 2011). However, recent behavioral experiments have suggested that people sometimes fail to notice mismatches between their intended choices and actual outcomes and even offer reasons for choices they never made (Johansson et al., 2005, 2008; Hall et al., 2010, 2012; Pärnamets et al., 2023). In the first report of “choice blindness” (CB), researchers had participants choose which of two faces they find more attractive and then asked them to describe the reasons for their selection (Johansson et al., 2005). Yet, in certain trials the experimenter used a sleight-of-hand technique to exchange the pictures, so that participants were presented with the non-selected picture as their intended choice. Surprisingly, participants called out and reported the manipulated outcomes only in 13% of the trials. Hence, in most manipulated trials they appeared to have accepted the non-selected choice as their own and presented reasons for it. Similar results have been observed using computerized paradigms (Johansson et al., 2008, 2014; Taya et al., 2014; Luo and Yu, 2017), auditory (Sauerland et al., 2013a), tactile (Steenfeldt-Kristensen and Thornton, 2013), gustatory and olfactory stimuli (Hall et al., 2010). Exposure to false outcomes has been reported to induce preference changes (Johansson et al., 2014), “distort” memories (Pärnamets et al., 2015), “shift political attitudes and voter intentions” (Hall et al., 2013), and “reverse [moral attitudes] moments after they were announced” (Hall et al., 2012).

These observations are generally presented as evidence that humans lack introspective access to their cognitive processes (as proposed by Nisbett and Wilson, 1977), and that our attitudes and preferences are less stable than commonly assumed (see e.g., Johansson and Hall, 2006; Hall et al., 2012; Johansson et al., 2014; Strandberg et al., 2020). Yet, CB is in stark contrast to common-sense intuition and experience of everyday life, in which we regularly notice outcomes that do not match our intentions (e.g., when receiving a wrong order in a restaurant). Moreover, it challenges established accounts of goal-directed behavior (Ridderinkhof et al., 2004; Walton et al., 2004; Ullsperger et al., 2014), learning (Schultz et al., 1997), agency and action (Frith et al., 2000; Alexander and Brown, 2011) that rest on internal representations of intentions and outcome monitoring. Are we so blind to our own intentions and beliefs that we would accept choices we never made and attitudes we disagree with as our own, and confabulate reasons for them?

**Figure 1:**
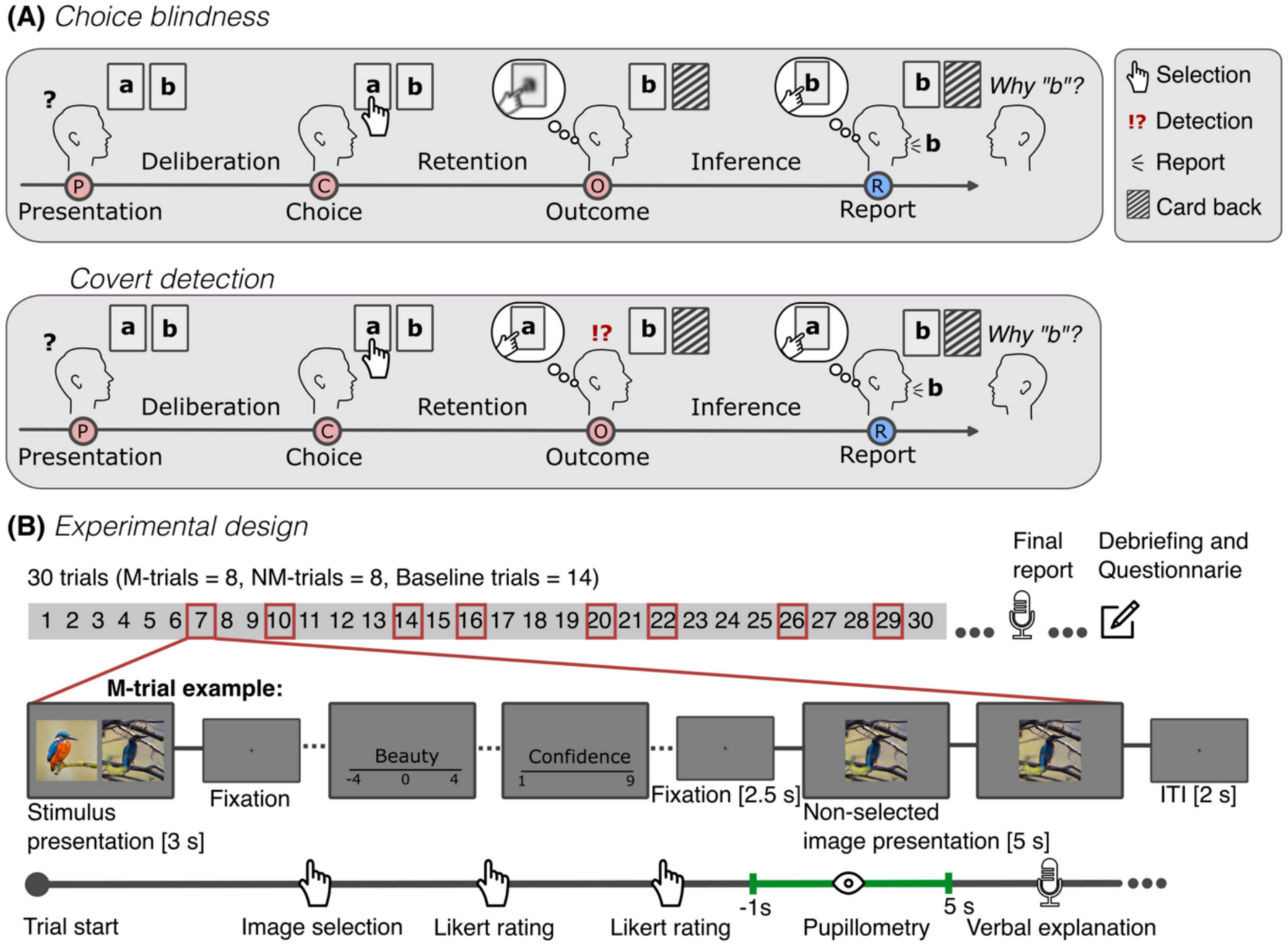
**(A)** Schematic visualization of CB and covert detection. In a CB task, participants are presented with two options (“a” and “b”), generally in card format, and asked to select one (presentation and choice). In manipulated trials (M-trials), after their selection (e.g., “a”), participants are presented with their non-selected option (“b”) as their choice (outcome) and asked to explain their decision (report). Often, instead of reporting this, participants simply provide reasons for the non-selected option (“b”). This reporting behavior is interpreted as evidence that participants did not notice the mismatch and is attributed to a form of introspective blindness to their decisions (visualized as blurred representation). In a covert detection, participants do likewise not protest in view of the mismatch and may even provide reasons for the non-selected option but do so while being aware that that was not their original choice. Thus, participants retain introspective knowledge about their decisions. **(B)** Overview of our experimental design and an example of a manipulation trial (M-trial). The experiment consisted of 30 trials, 8 of which were M-trials (Trial position 7, 10, 14, 16, 20, 22, 26, and 29). After 30 trials, participants were asked to report verbally if they noticed something special about the experiment (final report). Then, participants were debriefed and asked to fill-in the post-experiment questionnaire, with questions about their reporting behavior and a memory-based task. M- and NM-trials started with the presentation of a pair of images for 3 s. Participants were asked to select the image that they thought is considered more esthetically beautiful. Following the selection, participants were asked to rate how beautiful the image was, and how confident they are about their selection. After the ratings, participants were shown a fixation cross for 2.5 s and then their selected (NM-trials) or non-selected image (M-trials) was presented for 5 s as test image. To investigate pupil responses in view of the test image, analysis of pupil data was done during the period from 1 s before to 5 s after the test image presentation. After 5 s, participants were asked to verbally explain why they chose that image. The intertrial interval (ITI) was 2 s. Baseline trials consisted only of image selection, fixation and a 5 s presentation of the fixation. Pupil data from these trials were used as a baseline pupil response for expected outcomes. The photographs shown are for visualization only and were not used in the experiment.

Maybe not. First, CB experiments do not account for the fact that *detection* and *reporting* of wrong outcomes can substantially diverge. In CB studies, researchers rely on participants’ reports to determine whether a manipulated outcome was detected or not. Importantly, to avoid revealing the experimental aims to the participants, participants are not instructed or encouraged in any way to report mismatches. This leaves the possibility open that participants noticed the modified answers but did nothing to report them (Moore and Haggard, 2006, see Figure 1A). Moreover, many experiments are likely to result in low detection rates simply because of their designs, that often use strong real-world deception techniques such as magic tricks to manipulate responses (e.g., Johansson et al., 2005; Hall et al., 2010), as well as short presentation times and remarkably similar stimuli (e.g., Johansson et al., 2008, 2014; Pärnamets et al., 2020). If participants have no reasons to suspect a manipulation, a compelling magic trick may persuade them to believe the altered choice is theirs, since holding on to their original choice would mean questioning the workings of the physical world. Additionally, if participants notice that the experimenter is tricking them, they may feel reluctant to report this directly. When deciding between similar options and/or with low confidence, participants may also be prone to “change their mind” (Stone et al., 2022) or accept the false outcomes as their selection without providing any indication of detection due to indifference. Finally, choices in most previous CB experiments had no personal relevance and consequences whatsoever (but see Hall et al., 2010). We hypothesize that *stimulus discriminability* and *personal relevance* are likely to modulate both, detection and reporting in CB experiments.

Here, we conducted two experiments to test (1) if participants notice more manipulations than concurrently reported (*covert detection*), (2) if participants are more likely to notice and/or report more manipulations if the stimuli are more discriminable (*stimulus discriminability*), and (3) if participants are more likely to notice and report manipulations if the choices are more relevant to them (*personal relevance*).

In the first experiment, to reveal *covert detection*, we designed an eye-tracking CB paradigm to allow for pupillometry, included a retrospective memory task, and explicitly asked participants about their reporting behavior. The experiment had participants make simple esthetic choices of colored photographs on a computer and did not involve any real-world deception techniques. Participants were not informed about the manipulations but were given ample time to detect potential changes while undergoing pupil response measurements. This allowed to investigate whether pupil responses could serve as an objective physiological response that reflects differences between detected and undetected manipulated trials. Moreover, we tested if variations in *stimulus discriminability* (i.e., differences between esthetic beauty ratings of color photographs) modulated detection rates. Finally, we provided a subset of participants an *additional financial motivation* to investigate if detection rates were affected by the increased motivation. In the second experiment, we used monochromes females faces instead of color photographs to increase comparability to previous CB experiments (Johansson et al., 2005, 2008, 2014; Taya et al., 2014; Luo and Yu, 2017; Pärnamets et al., 2023) and had no additional financial motivation.

We present conclusive evidence that participants often noticed manipulated choices without reporting them and that the stimulus discriminability strongly affected CB. Hence, our results cast doubt on the ubiquity and behavioral relevance of previous CB studies and present a more cautious and intuitive interpretation of CB, in which participants are not as introspectively blind as originally portrayed.

## Materials and methods

### Subjects

We measured a total of 64 naïve participants in two CB experiments; 41 subjects participated in the first experiment (Exp. 1: 23 females, 17 males, 1 diverse; mean age ± SD 24.83 ± 4.81) and 23 in the second experiment (Exp. 2: 14 females, 8 males, 1 no answer, mean age ± SD 24.62 ± 4.67). The experiments were approved by ethic commission in psychological research of the University of Tübingen. Participants gave informed consent before and after debriefing on the experiment’s goals. They received either monetary compensation or course credits.

Note that due to missing or invalid eye tracking data the sample sizes for the behavioral and pupillometry analyses differ. In Exp. 1, 35 of the 41 participants from the behavioral analysis were included in the pupillometry analyses (one participant had no data and five had data of insufficient quality). In Exp. 2, 21 of the 23 from the behavioral analysis were included in the pupillometry analyses (one had no data, and one had data of insufficient quality). For details about these exclusions, see Section “Stimulus presentation and pupillometry” and Supplementary Tables S8, S9.

### Sample size and power analysis

Sample size was determined based on prior literature and is comparable to similar computerized CB experiments (Johansson et al., 2008; Sagana et al., 2014; Taya et al., 2014). Because the likelihood of detecting a manipulation increases with the number of presentations, most previous CB experiments kept the number of manipulated trials low (e.g., Johansson et al., 2005: three M-trials; Hall et al., 2010: two M-trials) and increased the number of participants instead. Here, we increased the number of M-trials per participant to 8 to maximize power for pupillometry analyses, while maintaining a sample size comparable to previous CB studies (Johansson et al., 2008; Sagana et al., 2014; Taya et al., 2014). Based on effect sizes calculated from publicly available data from a previous CB experiment with pupillometry (Pärnamets et al., 2023) and simulated data, we estimate that ca. 20 participants are sufficient to detect moderate pupil effects (Cohens d ≈ 0.6) with 80% power (see section “Sample size and statistical power for pupillometry analyses” in Supplementary material). Note that, as M-trials were classified post-hoc based on reporting behavior and the retrospective memory task, some conditions included only few participants (e.g., only 11 participants in Exp. 1 had data for the “no report” M-trial condition). Therefore, failure to detect differences in pupil responses between post-hoc classified conditions with few paired samples may result from limited statistical power (*n* < 16 and *n* < 12 correspond to <60% power for two- and one-sided tests, respectively).

### Experiment 1: CB of colored photographs

In our first experiment, we investigated three intuitive reasons that could explain the high rates of unreported manipulations in previous CB studies: *covert detection, stimulus discriminability* and *personal relevance.* While not central to CB, these factors may modulate detection and reporting behavior. Participants would exhibit covert detection if they noticed a manipulated choice but chose not to report it. In turn, low stimulus discriminability or low personal relevance may reduce detection and reporting rates, thereby increasing the apparent prevalence of CB.

#### Pupil dilation as a function of concurrent, retrospective or no report

To investigate the possibility of *covert detection* in CB we designed a computerized version of the CB paradigm (Johansson et al., 2005) that allowed for concurrent pupil size measurements. Participants were tasked to make esthetic judgments of colored photographs (see Figure 1B). Then, in a subset of trials, participants were either shown their selection (i.e., non-manipulated trials, *NM-trials*) or the participant’s selection was exchanged with the non-selected picture (i.e., “manipulation trials,” *M-trials*), and they were asked to justify their decision. Participants were not informed about the manipulations. To allow for clear pupil responses and to give participants time to notice the changes, test images were shown for 5 s before verbal report. After the experiment, participants were debriefed about the manipulations and asked to identify trials in which they were presented with the non-selected picture (retrospective memory task).

Our design allowed us to investigate pupil responses as a function of post-hoc classified reporting types: trials in which manipulations were detected and reported (concurrent report) and trials in which participants did not report a manipulation (no concurrent report). Based on the results of the retrospective memory task, manipulated trials that were not reported were classified as either probably detected but not reported during the experiment (i.e., only retrospectively reported) and probably undetected (i.e., neither concurrently not retrospectively reported). As pupil size is known to increase with arousal (Mathôt, 2018) and surprise (Preuschoff et al., 2011; Lavín et al., 2014), we expected to observe a relative increase in pupil size when participants detected a manipulation, irrespective of the participants’ reporting behavior.

Accordingly, we expected pupil dilation in manipulated trials (*M-trials*) that were concurrently reported to be larger compared to trials without manipulation (*NM-trials* and *baseline trials*, see below). Similarly, we expected pupil dilation in trials without concurrent report and in retrospectively reported M-trials to be larger than NM-trials without manipulation and not different to concurrently reported M-trials. If so, this would be indicative that trials without concurrent reports and/or retrospective reports were in fact concurrent detections. Finally, following the same rationale, in M-trials without concurrent nor retrospective report (i.e., trials which would generally be considered “no detections”), we expected pupil dilation to be smaller compared to detected M-trials and to see no difference to trials without manipulations.

#### Stimuli: similar versus distinct esthetic scores

Moreover, to investigate how *stimulus discriminability* modulates detection and reporting behavior, M-trials consisted of either pairs of photographs with similar esthetic ratings or with clearly different esthetic ratings. Stimulus discriminability was operationalized as the difference of the overall esthetic rating between the two displayed stimuli from a photograph database with different esthetic scores (Kang et al., 2020). We hypothesized that manipulation of choices with pictures of dissimilar esthetic ratings would be more likely to be detected and reported compared to trials with more esthetically similar pictures.

#### Personal relevance: financial compensation versus not

Finally, to investigate if *personal relevance* of the choices would affect detection rates, we performed the experiment in two groups of participants. One group received a financial motivation, while the other group did not. We expected that participants with financial motivation would detect and report manipulations more often than participants without additional financial motivation.

#### Experimental design

The CB experiment consisted of a total of 30 trials (see Figure 1B for an overview). In each trial participants were shown a pair of colored photographs and were asked to choose the picture which they thought was rated to be more beautiful in a photographic competition. The photographs were displayed for 3 s. The decision was recorded via key press and was temporally unrestricted. In a subset of 16 trials, we asked participants to perform two ratings: first, the esthetics of the chosen image on a Likert scale (i.e., “From −4 (not at all) to 4 (very beautiful), how beautiful is the image?”). Second, how confident they were that they had chosen the right photograph on a Likert scale from 1 to 9 [i.e., “From 1 (not at all) to 9 (very sure)]. After giving their answers via button presses, participants were asked to look at a central fixation cross for 2.5 s. After that either the image they selected (8 NM-trials) or the image they did not select (8 M-trials) was presented centrally behind the fixation cross for 5 s. The fixation cross ensured the measurement of reliable pupil responses. Participants were asked to verbally explain the reasons for selecting the present picture (“Please explain your choice of this image”). We recorded verbal reports during this period. The 8 M-trials were presented in a fixed position (7, 10, 14, 16, 20, 22, 26, 29). The manipulated images were randomly selected from a pool of 30 pairs of images. The remaining 8 trials in which participants had to explain their decision, but that were not manipulated (NM-trials), were presented at random positions. Baseline trials (14 trials) consisted only of image selection, fixation cross and a 5 s presentation of the selected image. An experimenter was in the room throughout the experiment. Importantly, participants were not informed about the manipulated trials, nor encouraged to monitor their selection.

#### Stimuli

We selected 30 pairs of photographs with a similar visual content from the Explainable Visual Esthetics (EVA) dataset (Kang et al., 2020), which consists of over 4,000 images with ratings of esthetic attributes and overall esthetic opinion scores. We separated the images in two groups: 15 pairs with a similar overall rating (difference in overall esthetic score 0.14 ± 0.2 SD) and 15 pairs with a less similar overall rating (rating difference 1.69 ± 0.44 SD). We included esthetic similarity to test if differences in the attribute of interest would influence the probability of detection in the manipulated trials. We counterbalanced the presented image pairs in the first M-trial across participants to ensure a balanced number of first M-trials showing similar and distant images pairs in our sample. Images were cropped to a square shape, and luminance- and contrast-matched in HSV-space using dedicated MATLAB code (for similar and dissimilar pair of images separately). Mean luminance and RMS-contrast from the brightness V channel (rescaled to 0–255) from the shown test images were 129.71 ± 0.55 and 59.72 ± 1.17, respectively, and had no difference between M- and NM-trials.

#### Motivation treatment and esthetic rating task

To test whether participants’ motivation affected detection rates, participants were separated into two groups: one with an extra financial motivation, and the other one without. All participants were told that the images selected were from an online photography contest and that each had an esthetic rating. Participants were asked to select the picture that they thought had received the higher rating. Participants in the motivation group (*n* = 21) were additionally told that each correct answer would increase their chance to participate in raffle for four 50 Euro coupons. In fact, all 41 participants were equally included in the raffle. Participants in the motivation group were provided immediate feedback (right/wrong) on their choices after the fixation phase of the first five trials. The remaining participants (*n* = 20) received no additional financial motivation and received no feedback during the first trials.

After the experiment, participants were asked to rate their motivation on a Likert scale (From 1 “not at all” to 7 “very motivated”). While motivation was significantly different between the groups (Wilcoxon rank sum test *W* = 282, *p* = 0.048), ratings were high in both groups (motivation group: 6.24 ± 0.77, neutral group: 5.6 ± 1.14).

#### Post-experiment questionnaire and debriefing

Directly after the experiment, participants were asked to verbally answer if they noticed something special about the experiment. Afterwards, we debriefed participants and asked them to fill in a short questionnaire. The questionnaire was designed to test for possible detections. First, they were asked: “Did you notice or suspect the manipulated responses? (Yes/mostly yes or No/mostly no). This was followed by: “If yes/mostly yes: How many manipulated responses did you notice/suspect?.” We further asked: “If you detected the manipulated responses, did you say anything about them during your explanations?” (Yes/mostly yes or No/mostly no). In case of a positive answer, they would be asked “How often did you say?.” In case of a negative answer, we asked them: “What was the reason of not saying anything about manipulations during the experiment?.” We offered them a series of answers: “I thought I pressed the wrong key”; “I thought something wrong happened with the computer/experiment”; “I avoided/hesitated saying so”; “I thought it was part of the experiment”; “I did not care”; “other.” Participants were allowed to select more than one answer. We further asked in case of a negative answer participants to explained how they answered: “What did you do?” (“I explained my original decision”; “I came up with some new explanations”; “both”; “do not know”). Then, to test for retrospective detections, we asked them to look at a separate sheet showing all pairs of images and to select the pairs of images that they think were manipulated. Participants were not informed about this memory-based task beforehand. Importantly, the sheet did not show the pairs in the order they were presented to avert mnemonic aids based on the trial order. Finally, we asked if they “have […] heard of the phenomenon choice blindness in the past?” (Yes/mostly yes or No/mostly no) and if so, if they “[…] realize/suspect that you were participating in a choice blindness experiment?” (Yes/mostly yes or No/mostly no).

In Exp. 1, none of the two participants that claimed to have heard about CB in the past suspected participating in a CB experiment. Hence, participants were not expecting to receive any false outcomes and were genuinely surprised when encountering one.

### Experiment 2: CB of monochrome faces

In the second experiment participants (*n* = 23, see above for details) were shown pairs of monochrome female faces with neutral expressions and asked to pick which picture they found more attractive (as in Johansson et al., 2005, 2008, 2014; Taya et al., 2014; Luo and Yu, 2017; Pärnamets et al., 2023). The experimental design was similar to that of Exp. 1. Participants had no time limit to provide their selection, but were shown the two pictures for 5 s. Thereafter, in a subset of trials (8 NM-trials and 8 M-trials), they were asked to rate the attractiveness and confidence in their selection using Likert scales and to verbally explain their choice. The experiment was followed by a post-experiment questionnaire that asked general detection questions and tested for retrospective report of manipulated choices. In Exp. 2, only one participant of the two participants who knew about CB suspected to be participating in a CB experiment.

The face stimuli and attractiveness ratings were taken from the Chicago Face Dataset (Ma et al., 2015, 2021). We selected 30 pairs of female faces with neutral expressions and matched ethnicity. Pictures shown in the eight M-trials were separated into four similar attractivity pairs (mean rating difference < 1) or four dissimilar attractivity pairs (mean rating difference > 1.5). Images were converted to grayscale and the foreground of the images were luminance matched using the SHINE toolbox for each image pair (Willenbockel et al., 2010). For the shown test images, the mean luminance was 112.73 ± 4.1 with a mean RMS-contrast of 57.53 ± 1.05. Luminance was comparable between M- and NM-trials (mean luminance difference: 1.74 ± 5.84; *t*(20) = 1.37, *p* = 0.19), while the RMS-contrast was marginally higher for M-trials (mean RMS-contrast in M-trials = 58.21; in NM-trials = 57.51; contrast difference: 0.7 ± 1.37; *t*(20) = 2.36, *p* = 0.03). Note that this small difference in RMS-contrast between M- and NM-trials is unlikely to have a systematic effect on the pupil response and would, if at all, increase pupil constriction in M-trials (against our expectations).

### Behavioral detection and reporting criteria

Two raters independently analyzed verbal reports to probe if participants detected manipulations (PRG and LH). Interrater agreement was high in both Experiments (Exp. 1: Cohen’s kappa = 0.94, percent agreement = 97%; Exp. 2: Cohen’s kappa = 83.2, percent agreement = 91.3%). Coding differences were solved by a joint re-assessment. Trials were categorized as “concurrent report” if participants showed any sign of mismatch detection in their verbal reports (e.g., “This is not my selection,” “I selected the other picture”). Moreover, explanations with a clear and explicit reference to the original (non-manipulated) selection were also coded as concurrent reports. Vague reports or indications of possible detection were flagged as “possible detection.” Verbal reports not including any sign of detection were considered as “no concurrent report.” Trials that were correctly remembered as manipulated in the post-experiment questionnaire were noted as “retrospective reports.” Trials with “possible detection” (in Exp 1: 5 out of 328 trials, in Exp 2: 6 out of 184 trials) that were not retrospectively reported were not further analyzed (in Exp. 1: 3 trials, in Exp. 2: 4 trials). Finally, “general detection” was defined based on the final question if they noticed something special about the experiment (before debriefing) and the first questionnaire item after debriefing (“Did you notice or suspect the manipulated responses?”).

Please note that despite a high interrater agreement, the interpretation and coding of participants’ spontaneous verbal responses lack an objective coding scheme and remains subjective. As an alternative to verbal reports, most computerized CB experiments allow participants to change their choice in M-trials via button press to identify detections (e.g., Pärnamets et al., 2023). However, using spontaneous verbal reports allows for a better understanding and judgment of participants behavior and awareness of the manipulations. To exemplify this, we present transcripts and corresponding coding from 96 unique M-trials from Exp. 1 (from 12 participants; 54 concurrently detected trials) and 10 from Exp. 2 (from 5 participants; 6 concurrently detected trials) in Supplementary Tables S2–S5. This analysis of verbal reports allowed for a clear identification of example cases of covert detection and conscious changes of mind, which would have been impossible otherwise.

### Stimulus presentation and pupillometry

The experiments were run with Psychtoolbox 3 (Brainard, 1997) in Matlab 2019a (MathWorks, Natick, MA) on a Linux computer and presented on a display with a resolution of 1,920 × 1,080. Images in Exp. 1 were 8 × 8° degrees of visual angle, while images in Exp. 2 were 12 × 5° degrees of visual angle.

Pupil size was measured monocularly throughout the experiments using an EyeLink 1,000 eye-tracker (SR-Research, Ottawa, Canada) at 1,000 Hz. Data preprocessing and analysis was performed using dedicated MATLAB code, following a pupillometry preprocessing pipeline outlined in Knapen et al. (2016) and Urai et al. (2017). Blinks and missing data identified by the EyeLink parser were linearly interpolated and padded by 150 ms. After interpolations, pupil data were filtered between 0.02 and 4 Hz using a third-order Butterworth filter, standardized (z-scored) to the pre-stimulus baseline (−1 s) and downsampled to 100 Hz. Blinks and saccades were regressed out from the pupil data using canonical pupil response functions (as described in Knapen et al., 2016). We constrained our analysis to the time around the presentation of the selected or manipulated images, where participants were asked to fixate the center of the screen (−1 to 5 s) and before they provided verbal reports. Trials were averaged per participant per condition (i.e., concurrent report, retrospective report, no report, M-, NM- and baseline trials). Note that trials with more than a third of invalid data points (i.e., 2 s) were excluded from the analysis. Participants with more than half of invalid trials (15 out of 30) were excluded from the eye tracking analysis (Exp. 1: 6 participants; Exp. 2: 1 participant). Moreover, pupil data from one participant could not be analyzed from Exp. 1 and we did not collect pupil data from one participant in Exp. 2. The total number of participants analyzed was *n* = 35 in Exp. 1 and *n* = 21 in Exp. 2. An overview of all available pupil trials per experimental condition (i.e., M-trials, NM-trials and baseline trials) and subject is shown in Supplementary Tables S8, S9.

### Data analysis and statistical inference

Behavioral data were analyzed using a mixed effects logistic regression using the “lmer” package (Bates et al., 2015) in R (v4.4.1, R Core Team, 2021). We modeled concurrent and retrospective report of the manipulated trials with trial order, confidence and image similarity as fixed factors and participants as a random factor using a maximum likelihood estimator in a mixed-effect logistic regression (Bates et al., 2015). We additionally included motivation as fixed factor for data from Exp. 1. Moreover, to test the ability to retrospectively discriminate M-trials from NM-trials and baseline trials, we report hit rate, false positive rate and the sensitivity index (d-prime) for retrospective reports.

Pupil-size time series were analyzed using paired *t*-tests with an FDR-correction for 0 to 5 s after the presentation of the test images. In case no time point survived FDR-correction, we report uncorrected values for the paired *t*-tests as exploratory analyses instead. Given our directional expectations that detection of manipulated choices in M-trials would enhance pupil dilation compared NM- and baseline trials, *t*-tests were conducted one-sided to increase statistical power, focusing the analyses on the predicted direction of the effects. Moreover, we extracted the average pupil responses from 2 to 4 s after the test image and normalized them with the baseline trials to perform a time-window analysis. We compared average pupil responses of M-trials, concurrently reported, not concurrently reported, retrospectively reported, as well as not reported M-trials against baseline (one-sided), NM-trials (one-sided) and each other (two-sided).

Furthermore, to account for the unequal number of trials and individual variability, we performed linear mixed models to predict average pupil size by trial type (M- vs. NM-trials) (model 1), to predict average pupil size in M-trials by reporting behavior (concurrently, retrospectively and not reported) (model 2) and trial order and confidence ratings (model 3). All models included participants as a random factor.

Finally, we tested whether mean pupil responses could classify trial conditions (M- vs. NM-trials) and concurrent report within M-trials using a threshold-based approach with leave-one-subject-out cross-validation. For trial condition classification, trials from one participant were classified based on whether their mean pupil response exceeded a threshold, defined as the midpoint of the mean response values for M- and NM-trials from all other participants. For concurrent detection in M-trials, trials were classified based on whether their mean pupil response exceed the midpoint of the mean values for concurrently reported and not concurrently reported M-trials from other participants. Statistical significance was assessed with binomial tests comparing accuracy against the majority class proportion.

## Results

### Experiment 1: CB of colored photographs

#### Detection and reporting behavior

**Figure 2:**
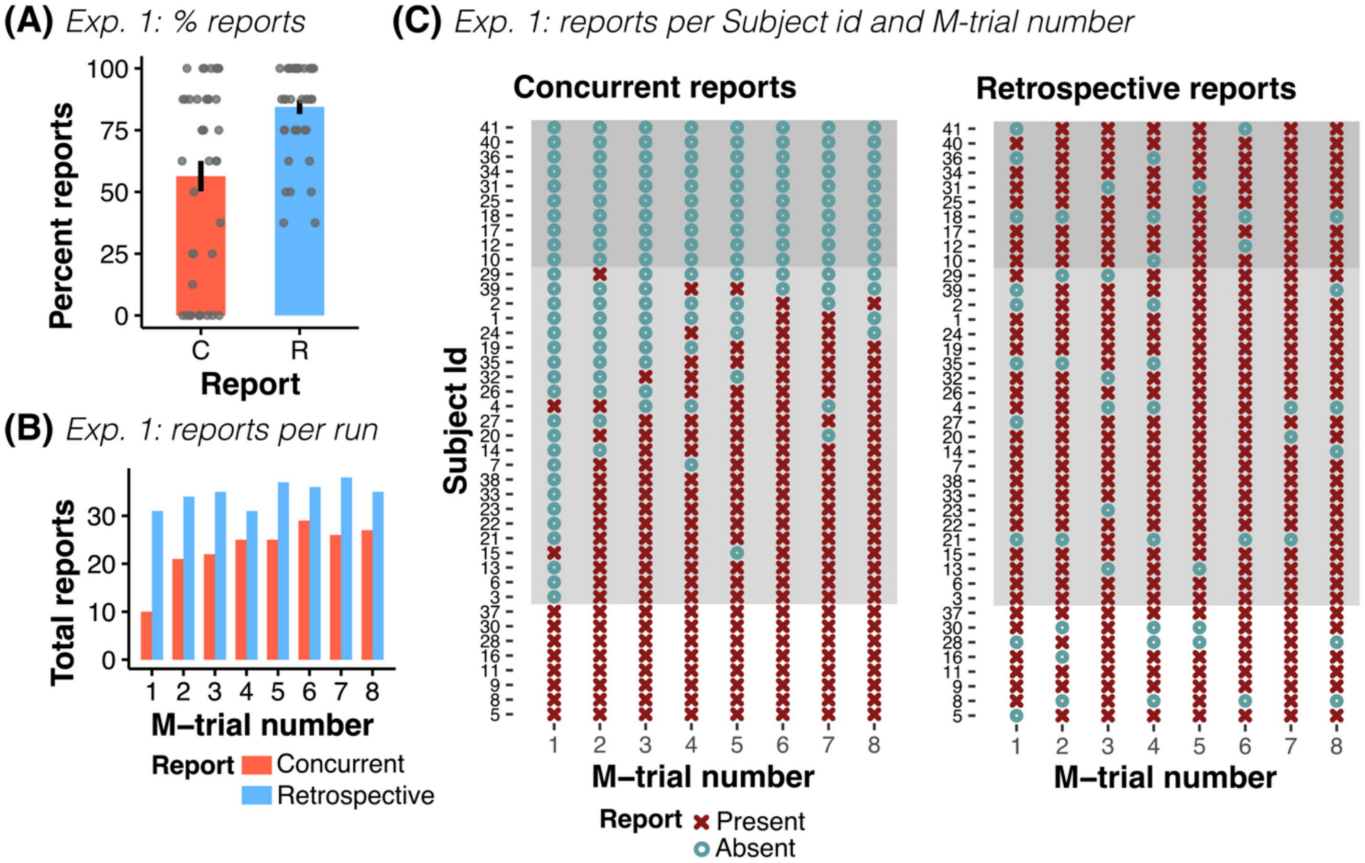
Experiment 1: Concurrent and retrospective reports. **(A)** Percent of concurrent (C) and retrospective (R) reports across all participants of Exp. 1. **(B)** Total number of concurrent and retrospective reports as a function of M-trial number (for *n* = 41 participants). **(C)** Overview of all participants’ responses, shown separately for reports during the experiment (concurrent) and for reports in the post-experiment questionnaire (retrospective), grouped by reporting behavior (no report, *n* = 10; some report, *n* = 23; all reports, *n* = 8). In participants with at least some reporting, the probability of reporting increased with trial order. While some participants did not concurrently report any (or only a few) manipulations, all participants reliably remembered manipulated trials, independent of their concurrent reporting behavior.

In general, detection of false outcomes in M-trials was remarkably high: together, participants concurrently and retrospectively reported 92.1% of all M-trials (302 out of 328 trials), while only 7% of the trials were neither concurrently nor retrospectively reported (23 trials). Participants concurrently reported 56.4% of manipulations (185 out of 328 trials, mean concurrent report was 4.51 ± 3.16 trials out of 8 M-trials), which is comparable to other computerized CB experiments (e.g., Pärnamets et al., 2015, 2023; Lachaud et al., 2022). After debriefing, however, participants correctly retrospectively identified 84.45% of the trials (277 out of 328 trials, mean retrospective report was 6.76 ± 1.5 trials out of 8 M-trials, see Figure 2A). On average, participants provided 7.49 ± 1.52 retrospective answers with a total of 90.2% of correct retrospective reports (277 out of 307 retrospective responses). From the concurrent reports, 86.5% were also retrospectively reported (160 of 185). Importantly, a total of 83.33% of the not concurrently reported M-trials were correctly retrospectively reported (115 of 135). Participants had an average of 0.731 ± 0.975 false positive retrospective responses. They were hence capable of discriminating M-trials from non-manipulated trials with a high hit rate (0.844 ± 0.187), low false positive rate (0.033 ± 0.044) and excellent sensitivity (d-prime = 2.728 ± 0.792).

All participants with no concurrent report (*n* = 10) retrospectively reported trials with an average of 6.7 ± 1.57 retrospective reports and no difference to participants with at least one concurrent report was observed (Welch’s unequal variances *t*-test: *t*(14.7) = −0.13, *p* = 0.897). Accordingly, we observed a stark contrast between concurrent and retrospective responses. For example, while only 10 of the 41 participants concurrently reported a false outcome in the first M-trial (24.4% of the participants), 31 remembered it retrospectively (75.6% of the participants, see Figure 2B).

Against our expectations, neither financial motivation of the participants nor image similarity affected reporting rates in Exp. 1 (OR_Motivation_ = 0.088, CI-95% [0.004, 1.72], *p* = 0.109; OR_Similarity_ = 0.658, CI-95% [0.28, 1.53], *p* = 0.33). We found that concurrent reporting rates increased with trial number (OR_Trialnr_ = 1.904, CI-95% [1.50, 2.41], *p* < 0.001) and confidence (OR_Confidence_ = 1.478, CI-95% [1.13, 1.93], *p* = 0.004). However, this relationship was weaker for retrospective reports (OR_Trialnr_ = 1.18, CI-95% [1.02, 1.37], *p* = 0.03, and OR_Confidence_ = 1.198, CI-95% [0.99, 1.45], *p* = 0.06, see Supplementary Figure S2).

Together, these behavioral results show that most participants in fact *detected manipulations* but *did not report them* during the experiment.

#### Questionnaire responses and self-reported behavior

Before debriefing, participants were asked if they noticed anything special about the experiment. Only 4 of the 41 participants said nothing about the manipulations (three of which had concurrently reported manipulations), while 30 reported the selection changes. Seven participants closed the experiment without providing a recorded answer to the question but reported noticing the changes during the debriefing (a transcript of all provided answers can be seen in Supplementary Table S1). After debriefing, all participants claimed to have noticed the false outcomes. Why did some participants (*n* = 10) then fail to report the detections during the experiment? Interestingly, most of the participants with at least some concurrent report (11 of 13), answered they said nothing because they thought they pressed the wrong key, or it was an error of the experiment (7 of 13), while 9 out of the 10 participants without any concurrent report said they thought false outcomes were part of the experiment (see Supplementary Figure S3). For example, one participant, who did not give any indication of detection during the explanation phase, was noticeably surprised when the non-selected image appeared in the first two M-trials (reacting with expressions such as “Huh? I did not choose that”). However, they then simply proceeded to provide reasons for a choice they had not made, assuming it was part of the experiment.

Moreover, and consistent with the behavioral results, 8 out of the 10 participants without any concurrent report said they provided reasons for selecting the manipulated choices, i.e., they accepted the false outcome and confabulated reasons for the provided image, and all 10 confirmed that they provided no report. Finally, participants were remarkably good at estimating how many trials were manipulated (mean 7.06 ± 1.54 SD) and in describing their reporting behaviors (mean estimation of provided concurrent reports was 4.2 ± 3.18; mean difference to behavioral responses was only 0.25 ± 1.15).

#### Types of reporting behavior and examples of spontaneous reports

Our results show that participants did not display homogeneous reporting behavior: some participants noticed all manipulations and reported them directly (*n* = 8, see Figure 2C, see examples in Supplementary Table S2). Others noticed many manipulations but reported only some of them and often later in time (*n* = 23, see examples in Supplementary Table S3). Other participants noticed many manipulations but said nothing about them when explaining their answers (*n* = 10, see examples in Supplementary Table S4). The latter two reporting behaviors are particularly worrisome for CB experiments as they suggest that up to 80.5% of our participants exhibited covert detection (33 out of 41 participants).

Importantly, many participants described this behavior already during the experiment: “*Just a quick question. I’m pretty sure I selected the other image. Sometimes I thought I had chosen the other one and wasn’t sure if I was wrong, but this time I’m very sure […]*” (s01, M6, Trial 22), “*I have a question. I’m pretty sure that this is the second time I’ve seen a picture that I did not select. […]*” (s33, M2, Trial 10), *“[…] It has happened twice now that I selected a different picture […]”* (s38, M2, Trial 10) (see Supplementary Table S3). Similarly, even participants without any concurrent report describe covert detection just before debriefing: “*Some of the pictures I selected with the keyboard were not the ones that were displayed.”* (s10)*, “Yes, that the images I chose were not the ones I was asked why I chose them.”* (s12), *“From time to time I was shown the wrong picture that I had not selected.”* (s41) (see all responses in Supplementary Table S1).

Moreover, participants often provided verbal explanations that were indicative of indifference and/or explicit “changes of mind” in CB: “*I thought the images were similar, so I just selected one*.” (s20, M1, Trial 7, manipulation was correctly retrospectively reported), “*I think both pictures look similar but then this one - it looks more vivid.*” (s29, M4, Trial 16, correct retrospective report), “*I think both of these images were actually quite beautiful, but maybe people would choose this above the other because it is technically quite nice.*” (s22, M1, Trial 7, correct retrospective report), “*The pictures were very similar. It was a 50:50 decision*.” (s27, M2, Trial 10, correct retrospective report), *“The choice was difficult for me because the pictures look very similar […]”* (s04, M4, Trial 16, correct retrospective report).

#### Pupil-responses

**Figure 3:**
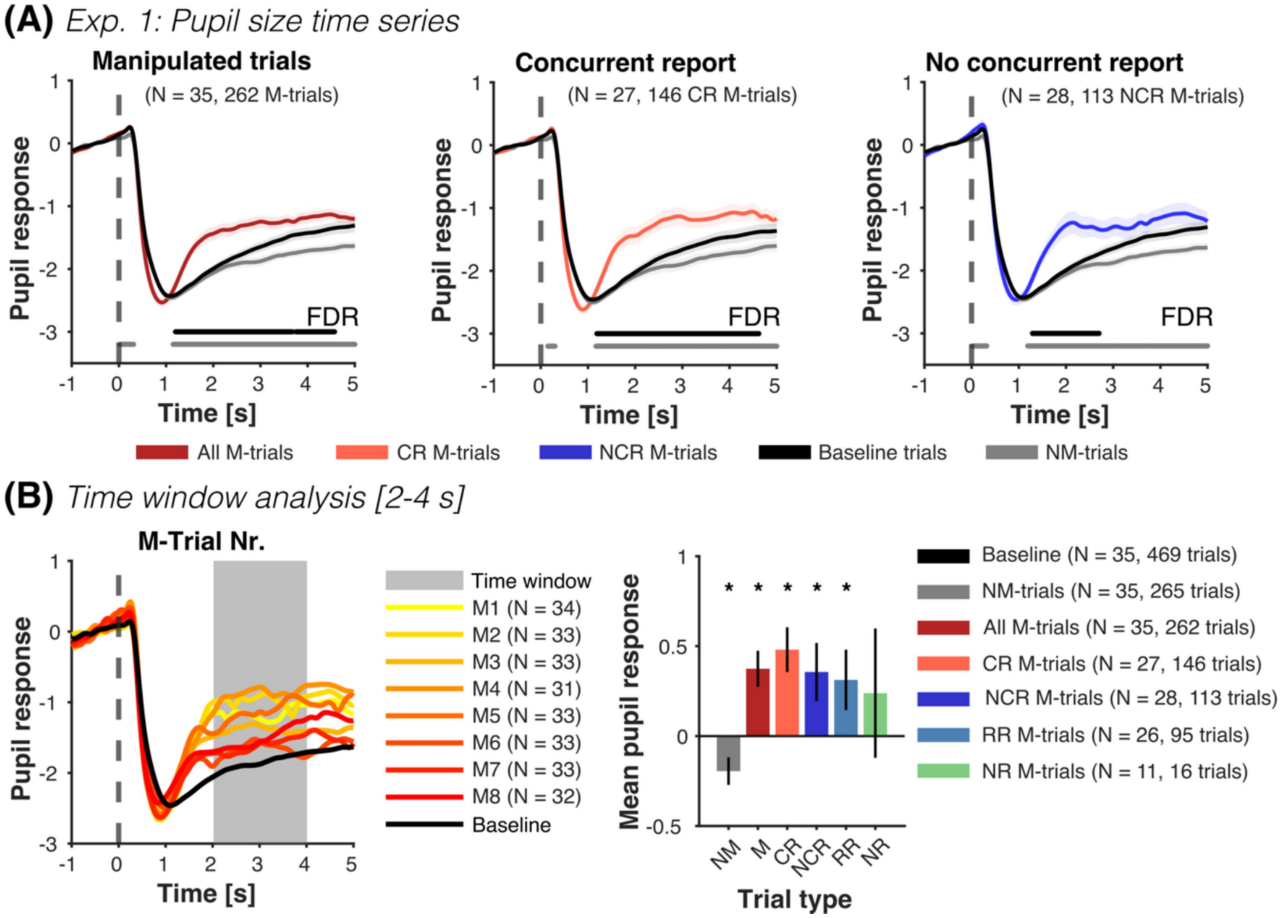
Experiment 1: Pupil size as a function of manipulated trials and reports. **(A)** Mean pupil size time series (±SEM) for all M-trials (dark red), concurrent report (red) and no concurrent report (blue), along with NM-trials (gray) and baseline trials (black). Horizontal lines parallel to x-axis show significant differences (one-sided paired *t*-tests, FDR-corrected) to NM-trials (gray line) and baseline trials (black line). Manipulated test images from M-trials that were reported concurrently and not concurrently reported elicited an increase in pupil size compared to NM and baseline trials. Pupil responses in trials without concurrent report reflect mainly retrospectively reported trials (see Supplementary Figure S5A). Time series of M-trials with no report were only moderately higher than the NM-trials later in time (see Supplementary Figure S5B). **(B)** In the time windows analysis, we extracted the mean pupil response from 2 to 4 s after the presentation of the test image and normalized it with the 14 baseline trials per subject (left plot shows the mean pupil time series of all included participants). M-trials (M), concurrent reported (CR), not concurrently reported (NCR), and retrospectively reported (RR) were significantly different from baseline and NM trials (right plot, one-sided paired *t*-tests, see Supplementary Table S6). No-report M-trials (NR) were only numerically so. Pupil responses in NM-trials were significantly smaller than in baseline trials (two-sided paired *t*-test). Finally, we observed no difference between the different M-trials (concurrent, not concurrent, retrospective and no-report) (all two-sided paired *t*-tests *p* > 0.05). For each figure, we report the number of participants, as well as the total number of trials contributing to each condition in parenthesis. *p < 0.05.

To further test covert detection, we analyzed pupil size time series during the presentation of the test images. As hypothesized, pupil responses during M-trials and during the concurrent report of false outcomes elicited a significant increase in pupil size when compared to baseline and NM-trials (see Figure 3A, left and middle; plots for individual subjects are shown in Supplementary Figure S4). Remarkably, pupil size in trials without concurrent report (i.e., including retrospective and no-report M-trials) was also significantly larger than baseline and NM-trials (see Figure 3A, right), especially in those retrospectively reported as M-trials (see Supplementary Figure S5A).

Along the same line, pupil responses averaged from 2 to 4 s after test image presentation show that reported M-trials were significantly larger than baseline and NM-trials (see Figure 3B and Supplementary Table S6). Intriguingly, NM-trials were significantly smaller than baseline trials.

Time-series and averaged values of pupil responses during no-report M-trials were only marginally larger than normal NM-trials (see Figure 3B and Supplementary Figure S5B) and not different to other M-trials. However, please note that the reliability of these estimates is limited by the small number of available pupil traces from no report M-trials (*N* = 11, with a total of 16 trials).

Accordingly, a linear mixed-model for trial condition revealed a significant increase in pupil size in M-trials compared to NM-trials (β_M-Trial_ = 0.576, CI-95% [0.446, 0.706], *p* < 0.001). Similarly, a linear mixed-model with classified M-trials (concurrently reported, retrospectively reported and no-report), showed that concurrently reported and retrospectively reported M-trials were larger than NM-trials (β_CR_ = 0.645, CI-95% [0.484, 0.806], *p* < 0.001; β_RR_ = 0.529, CI-95% [0.34, 0.719], *p* < 0.001). This increase was not observed for no-report M-trials (β_NR_ = 0.248, CI-95% [−0.112, 0.607], *p* = 0.177). A final model showed that pupil size decreased with M-trial order (β_Trialnr_ = −0.077, CI-95% [−0.122, −0.033], *p* < 0.001), but was not influenced by confidence (β_Confidence_ = 0.046, CI-95% [−0.01, 0.1], *p* = 0.112). The variance of the random intercept across models was similar (0.181, 0.175, 0.252), with ca. 23–25% of the total variance explained by between-subject differences (ICC = 0.238, 0.232, 0.266).

Finally, we tested whether we could classify trial condition (M- vs. NM-trials) and concurrent detection within M-trials using a threshold-based approach with leave-one-subject-out cross-validation. Pupil responses accurately classified trial conditions (345/527 trials correct, 65.5% accuracy, *p* < 0.001, binomial test against class majority proportion). However, they did not predict concurrent report within M-trials above chance (133/259 trials correct, 51.4% accuracy, *p* = 0.95).

Thus, pupil responses show, in accord to detection and reporting behavior, that many participants noticed false outcomes, but did not report them: pupil responses in concurrently reported, not concurrently reported and retrospectively reported M-trials were significantly different to baseline and NM-trials without manipulations (see Supplementary Table S6) and not different to each other.

### Experiment 2: CB of monochrome faces

#### Detection and reporting behavior

In the second experiment, we used monochrome photographs of faces. This allowed us to test for potential differences to the colored photographs used in Exp. 1 and compare previous CB experiments using faces (Johansson et al., 2005, 2008, 2014; Taya et al., 2014; Luo and Yu, 2017).

**Figure 4:**
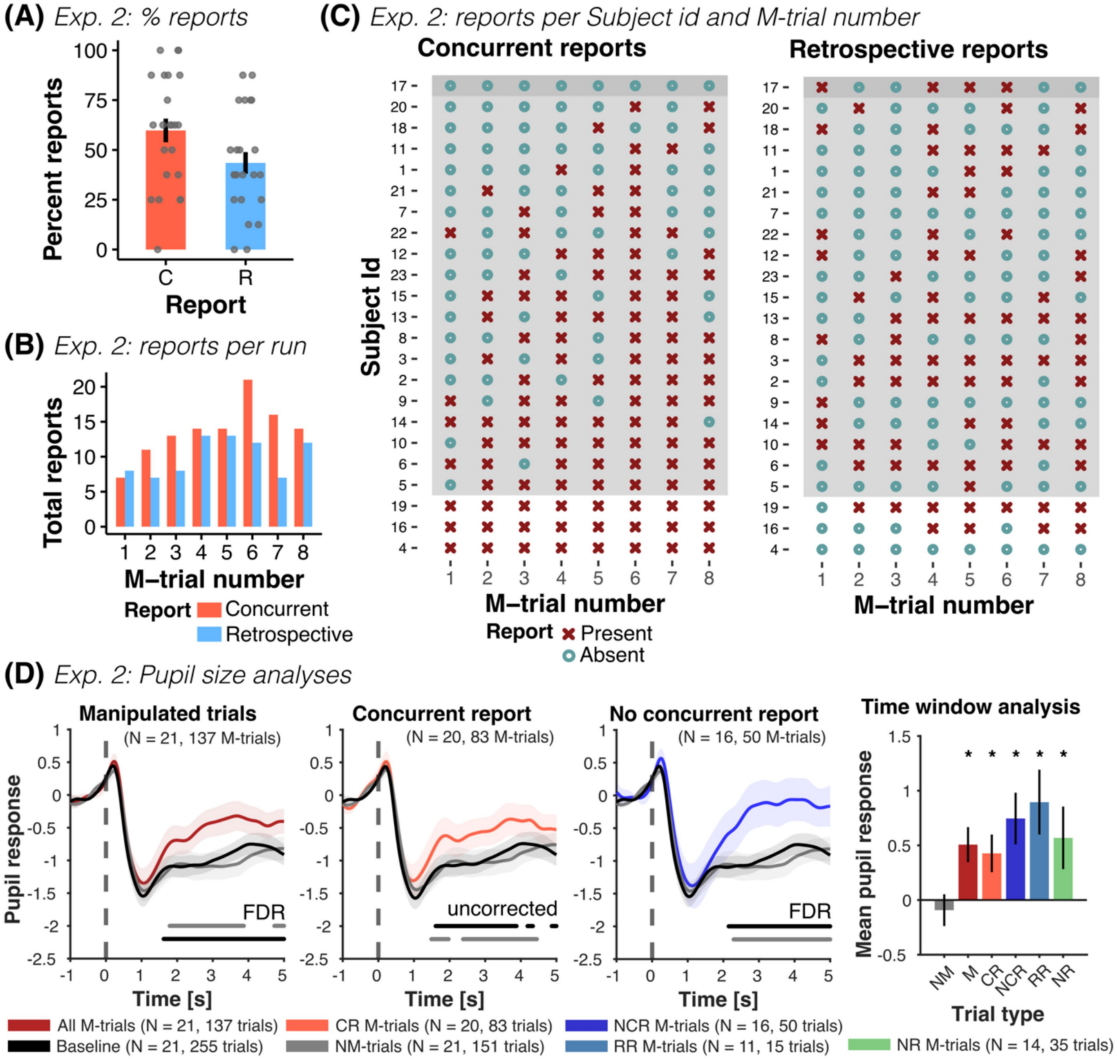
Experiment 2: Concurrent and retrospective reports and pupil size analyses. **(A)** Percent of concurrent (C) and retrospective (R) reports across all participants of Exp. 2. **(B)** Total number of concurrent and retrospective reports as a function of M-trial number (for *n* = 23 participants). **(C)** Overview of all participants’ responses, shown separately for reports during the experiment (concurrent) and for reports in the post-experiment questionnaire (retrospective), grouped by reporting behavior (no report, *n* = 1; some report, *n* = 19; all reports, *n* = 3). In participants with at least some reporting, the probability of reporting increased with trial order. **(D)** Mean pupil size time series (± SEM) for all M-trials (dark red), concurrent report (red) and no concurrent report (blue), along with NM-trials (gray) and baseline trials (black). Horizontal lines parallel to x-axis show significant differences (one-sided paired *t*-tests, FDR-corrected or uncorrected) to NM-trials (gray line) and baseline trials (black line). M-trials with and without concurrent report elicited an increase in pupil size compared to NM and baseline trials. Time series of M-trials with retrospective reports and with no report were also higher than baseline and NM-trials (see Supplementary Figures S5A,B). The time-window analysis of the mean pupil response from 2 to 4 s after the presentation of the test image showed the same pattern of results: mean pupil responses in manipulated trials (M: all M-trials; CR: M-trials with concurrent report; NCR: M-trials without concurrent report; RR: M-trials with retrospective report only and NR: M-trials with no report) were consistently larger than NM and baseline trials, irrespective of reporting behavior (one-sided paired *t*-tests). NM-trials were not different to baseline trials (two-sided paired *t*-test). Finally, we observed no difference between the different M-trials (all two-sided paired *t*-tests > 0.05), with exception of retrospective report compared to no report trials (see Supplementary Table S7). For each figure, we report the number of participants, as well as the total number of trials contributing to each condition in parenthesis. *p < 0.05.

Participants showed a relatively high concurrent and retrospective report: overall 71.7% of the trials were concurrently and retrospectively reported (132 out of 184 trials), while roughly a fourth of the trials remained unreported (26.1%, 48 out of 184 trials) (see Figure 4A). Participants concurrently reported 59.78% of the false outcomes (110 out of 184 trials, mean concurrent report was 4.78 ± 2.275 out of the 8 M-trials). This reporting rate is comparable to Exp. 1 and previous computerized CB studies (e.g., Pärnamets et al., 2015, 2023; Lachaud et al., 2022). Only 43.47% of the M-trials were retrospectively reported (80 out of 184 trials, mean correct retrospective report was 3.48 ± 2.09 out of the 8 M-trials). On average, participants provided 5.39 ± 3.61 retrospective reports, with a total of 64.5% correct retrospective reports (i.e., 80 out of 124 retrospective reports), and an average of 1.913 ± 2.592 false positive retrospective responses. Moreover, only 52.7% of the concurrently reported M-trials were retrospectively reported (in Exp. 1: 86.5%). In turn, 29.4% of the not concurrently reported M-trials were correctly retrospectively reported (20 out of 68, in Exp. 1: 83.33%). Thus, participants were capable of retrospectively discriminating the change selection of monochrome faces, with a hit rate of 0.435 ± 0.261, false positive rate of 0.087 ± 0.118 and moderate sensitivity (d-prime = 1.231 ± 0.851). However, their performance was substantially lower than in Exp. 1 (d-prime = 2.729 ± 0.792), indicating a considerable reduction in retrospective discrimination ability when using monochrome faces (see noticeable differences in Figure 4C).

Reporting rates increased with the number of M-trials (OR_Trialnr_ = 1.508, CI-95% [1.227, 1.852], *p* < 0.001) and with the reported confidence in the selection (OR_Confidence_ = 1.725, CI-95% [1.227, 2.424], *p* = 0.002), in accord to Exp. 1 (see Figure 4B). This relationship disappeared for retrospective reports (OR_Trialnr_ = 1.118, CI-95% [0.967, 1.291], *p* = 0.1315 and OR_Confidence_ = 1.04, CI-95% [0.846, 1.28], *p* = 0.706). Moreover, the attractiveness similarity of the images significantly affected the concurrent report rate: images with a similar attractiveness rating were reported less often, compared to more dissimilar images (OR_Similarity_ = 0.283, CI-95% [0.112, 0.711], *p* = 0.007). Finally, similar images were remembered less often as false outcomes in the post-experiment questionnaire (OR_Similarity_ = 0.483, CI-95% [0.236, 0.986], *p* = 0.046). Selected examples of verbal reports showing a difference between low- and high-confidence choices, indifference and “changes of mind” can be seen in Supplementary Table S5.

#### General detection

While roughly half of the participants reported the false outcome before debriefing (11 out of 20 participants), all participants (that answered the question, *n* = 22) claimed to have detected the changes after debriefing. This is indicative that many participants thought the manipulations were part of the experiment.

#### Pupillometry

Pupil responses were similar to those of Exp. 1: pupil size during concurrent and not concurrently reported M-trials were larger compared to baseline and NM-trials (see Figure 4D and Supplementary Table S7). Moreover, pupil responses in M-trials with retrospective reports and without concurrent nor retrospective report (i.e., no-report M-trials) were also larger than baseline and NM-trials and not different to trials with concurrent reports (see Supplementary Figures S5A,B and Supplementary Table S7). In contrast to Exp. 1, pupil responses in NM-trials were comparable to baseline trials. Linear mixed models revealed increased pupil responses in M-trials compared to NM-trials (β_M-Trial_ = 0.56, CI-95% [0.31, 0.808], *p* < 0.001), irrespective of reporting behavior (β_CR_ = 0.52, CI-95% [0.23, 0.816], *p* < 0.001; β_RR_ = 0.85, CI-95% [0.269, 1.431], *p* = 0.004, β_NR_ = 0.53, CI-95% [0.13, 0.922], *p* = 0.01). Pupil responses were not affected by trial order (β_Trialnr_ = −0.016, CI-95% [−0.093, 0.061], *p* = 0.69) nor confidence (β_Confidence_ = 0.007, CI-95% [−0.101, 0.115], *p* = 0.9). Across models the between-subject variance was similar (0.201, 0.205, 0.269) and accounted for 15–19% of the total variance (ICC = 0.15, 0.151, 0.195).

Finally, while classification of trial conditions (M- vs. NM-trials) based of mean pupil responses was possible (175/288 trials correct, 60.8% accuracy, *p* = 0.003, binomial test against class majority proportion), pupil responses did not predict concurrent report (61/133 trials correct, 45.9% accuracy, *p* = 1).”

## Discussion

In “choice blindness” (CB) tasks, participants are presented with an outcome that does not match their intentions. Prior literature largely suggests that participants seem not to notice the false outcome, accept it as their own choice and even present reasons for it. These observations have led to the conclusion that humans may have less knowledge about their intentions and attitudes than common-sense suggests (Johansson et al., 2005).

Here, we report behavioral and pupillometry data from two computerized CB experiments specifically designed to uncover initially non-reported detections of manipulated trials. The results reveal that when presented with false outcomes in a CB task, participants frequently detect the manipulations, but do not report them (i.e., covert detection). Thus, while we observed similar report rates to comparable computerized experiments (e.g., Pärnamets et al., 2015, 2023; Lachaud et al., 2022), our experiments show that when participants do not complain in view of a false outcome, this is frequently not because they fail to notice and are “choice blind.” Rather, many participants (up to 80%) withhold reporting for various reasons (they thought they pressed the wrong button or that it was part of the experiment, they were unsure about their decision, they changed their mind, etc.). Moreover, we show that when participants made low confidence decisions and when the options were similar, manipulations were less likely to be reported (albeit often detected). Below, we discuss our results in detail and tentatively conclude that previous observations of CB may be more parsimoniously explained by a combination of common-sense causes, rather than by assuming that participants were “choice blind.”

### Covert detection and reporting behavior in choice blindness

Previous CB research have made an effort to rule out covert detection, by either allowing participants to look over the stimuli after debriefing again (i.e., allowing for retrospective reports), providing indirect measurements, such as correlating questionnaire-based measures of social desirability and compliance with participants responses (with mixed results, cf. Sauerland et al., 2013a, 2013b; Aardema et al., 2014) or discussing how unlikely this would be in view of the participants reactions after debriefing (e.g., Johansson et al., 2005; Hall and Johansson, 2006; Hall et al., 2010).

However, all these provide only indirect evidence against covert detection. Here, we included a memory-based task and explicitly asked participants about the reasons not to report detected trials instead. They consistently provided different common-sense reasons that were compatible with their reporting behavior: most participants with at least some concurrent report initially thought they pressed the wrong key or that the experiment contained errors (see examples in Supplementary Tables S2, S3). In turn, participants without any concurrent report consistently thought the mismatches were part of the experiment and simply accepted the modified answers. Consistent with covert detection, all these participants correctly identified manipulated trials retrospectively.

Importantly, we observed that some participants were initially hesitant to report the errors, probably because they had to actively disengage from the task, question the workings of the computer experiment and describe the error to the researcher. This observation is particularly worrisome for CB experiments involving a direct social interaction between experimenter and participants (e.g., Johansson et al., 2005, 2014; Hall et al., 2010), as complaining about the false outcome entails signaling distrust and questioning the experimenter. This is something participants may be reluctant to do (cf. “accusatory reluctance,” see Ekman et al., 1980; O’Sullivan, 2003).

Overall, our results show that up to 80% of the participants were reluctant to report detected manipulations and were more likely to do so later in time. Moreover, across both experiments we estimate that 65.5% of all unreported M-trials were in fact covert detections (135 of 206; from Exp. 1: 115 of 138; from Exp. 2: 20 of 68). Many participants seemingly exhibiting CB were in fact correctly monitoring their responses and waited to collect enough information to correctly assess the source of the errors. Others simply thought the manipulations were part of the experiment and accepted the false outcomes. This observation provides conclusive evidence that covert detection may be common in (computerized) CB experiments: participants often notice, but do not report manipulated trials. This presents a serious challenge to CB, as it suggests that many participants deemed to be “choice blind” may in fact only withhold reporting.

Crucially, our results reflect participants behavior in *in-person computerized* CB tasks and may not fully apply to *non-computerized* experiments using deception techniques (such as magic tricks, e.g., Johansson et al., 2005; Hall et al., 2010, 2012, 2013). For example, the specific reasons for not reporting a detection are likely to differ: participants in non-computerized experiments may not refrain from reporting because they attribute deceptive manipulations to errors in the experiment or simply see them as part of the experiment (as shown here), but rather because they assume they misremembered or feel reluctant to signal distrust. However, we see no compelling reason covert detection should be unique to in person computerized tasks, as error monitoring and mismatch detection should operate similarly across both settings. Hence, while the reasons for not reporting detections may differ, (covert) detections are likely present across both experiment forms.

### Pupil responses signal surprise in manipulated trials

Our pupillometry results add further support to the existence of covert detection in CB. They reveal increased pupil responses during manipulated trials, regardless of whether reports were made concurrently or not, with no difference between them. This increase in pupil size is consistent with reports of pupil dilation in view of unexpected events (Preuschoff et al., 2011; Lavín et al., 2014) and the observation that participants noticed the false outcomes, even in absence of a verbal report. Moreover, pupil responses in manipulated trials were smaller with increased trial order, suggesting that participants were less surprised after repeated viewing of false outcomes (in Exp. 1). Accordingly, a recent fMRI experiment using a CB paradigm (Vogel et al., 2024) showed that manipulated trials evoked activity in brain regions involved in the processing of error monitoring and surprise (e.g., the anterior cingulate cortex, anterior insula and caudate nucleus) (Fouragnan et al., 2018; Alexander and Brown, 2019; Plikat et al., 2025).

A recent CB eye-tracking study also found that pupil size increased in all M-trials, including those not reported by participants, relative to NM-trials (Pärnamets et al., 2023). They further showed that detected M-trials had larger pupil responses compared to unreported M-trials (in contrast to our results). Yet, instead of interpreting these results as evidence that participants in fact noticed the manipulations in the unreported M-trials (with pupil dilation signaling arousal and surprise), the authors concluded that the increase in pupil size possibly reflects an increase in cognitive effort and that participants were unaware of the manipulations. Could the significant pupil dilation in unreported M-trials they report and that we also observe in M-trials that were not concurrently reported reflect something like an unconscious conflict?

This interpretation is unlikely in view of our behavioral and pupillometry results. First, our data suggest that many participants noticed the manipulations and either went along with them or modified their choices in response to the alternative outcome (i.e., they “changed their mind”). In fact, in both of our experiments nearly all participants reported noticing the manipulations when asked afterwards, many commented on them already during the experiment and correctly identified the manipulated trials afterwards. This is evidence that participants were often aware of the manipulations, even if they did not concurrently report them. Since Pärnamets et al. (2023) did not include a retrospective memory task, pupil dilation in unreported M-trials likely reflect a mix of genuinely unnoticed manipulations and manipulations that were noticed but not reported concurrently, as shown here. Finally, in the study from Pärnamets et al. (2023), participants not only showed increased pupil responses in accepted M-trials but also had longer response times than in NM-trials and lower confidence compared to corrected M-trials. Consistent with our observations, these results may also indicate some level of awareness about the manipulations.

Together, results of our and prior studies show that pupil responses differ between M-trials and NM-trials, and our results strongly suggest that pupil responses provide an objective indication of covert detection in CB. Pupil responses reveal participants’ surprise in view of the unexpected outcomes, irrespective of the subsequent reporting behavior.

### Stimulus properties and discriminability

Unsurprisingly, the idiosyncrasies of the stimulus used strongly modulated report rates: it was easier for participants to remember dissimilar faces (in Exp. 2) and to retrospectively remember manipulated trials of colored photographs (of Exp. 1) than to remember manipulations out of the 30 pairs of monochrome front portraits of female faces with neutral expressions (of Exp. 2). This is in accord with previous studies showing that stimulus discriminability facilitates reporting of M-trials, using visual (e.g., Somerville and McGowan, 2016), auditory (Sauerland et al., 2013a), gustatory and olfactory (Hall et al., 2010), and tactile (Steenfeldt-Kristensen and Thornton, 2013) stimuli. The low retrospective report rates we observed when using monochrome faces (in Exp. 2) are consistent with the observation that participants are remarkably bad at unfamiliar face identification (Jenkins and Burton, 2011; Sauerland et al., 2016) and remembering faces (Luo and Yu, 2017). Hence, CB in previous experiments using monochrome faces (e.g., Johansson et al., 2005, 2008, 2014; Taya et al., 2014; Pärnamets et al., 2020, 2023) may in part be attributable to the use of unfamiliar faces and not be generalizable to other visual stimuli. Most importantly, as the simple use of colored photographs with different content was sufficient to almost completely abolish CB, our results show that perceptual and mnemonic factors play in fact an important role in CB. We expect CB to be weaker/absent when using familiar faces.

### Motivation, familiarity, and personal relevance

Intuitively, we expect participants to notice manipulations if they care about the choices they make. Our results failed to reveal such an effect when providing a financial incentive (i.e., an extrinsic motivation). Yet, previous studies do suggest that familiarity and personal relevance modulate detection frequency. For example, when children were asked to pick between familiar chocolate brands, they easily detected false outcome trials (ca. 85% detections) (Somerville and McGowan, 2016), when participants were presented with false outcomes in a questionnaire about own norm-violation behaviors (e.g., cheating in an exam) they detected almost all changes (Sauerland et al., 2013b), and when participants were presented with false outcomes about things they had a strong attitude toward, they were more likely to detect changes compared to trials about things they did not care about (Rieznik et al., 2017; Strandberg et al., 2018). These results suggest that people are less susceptible to CB when decisions (intrinsically) matter for them. Our failure to influence detection rates by our motivation treatment in Exp. 1 may have resulted either from the different nature of a financial motivation compared to strong prior opinions about choices, or from the generally high detection rates and high motivation of participants to perform the task. Future studies investigating the role of motivation in CB should carefully design more effective ways of inducing motivation differences (e.g., by including incentives, cf. Hall et al., 2010).

### Confidence and evidence of “changes of mind”

Finally, we observed that when participants were confident about their decisions, the probability of reporting manipulations increased (as in Sauerland et al., 2016; Strandberg et al., 2019; Pärnamets et al., 2023). Conversely, when participants were unsure about their selection, they were less likely to report a manipulation. This indicates that participants were well aware and knowledgeable about their choices and attitudes. Decision uncertainty and low confidence are known to promote “changes of mind” (Stone et al., 2022). Indeed, some verbal reports of the participants indicate that they were often indifferent to the choices and prone to change their mind in view of the manipulated outcomes. It is hence possible that many participants who appeared “choice blind*”* have in fact changed their choice and were not introspectively blind (Bortolotti and Sullivan-Bissett, 2021). Our results show that participants were likely aware about these changes (in contrast to the interpretation of Bortolotti and Sullivan-Bissett, 2021), as the relationship between confidence and reporting behavior was absent in the retrospective reports: participants noticed the changes, even in low-confidence decisions, they just sometimes did not report them during the experiment.

Hence, the presentation of false outcomes in CB paradigms when participants are indifferent or unconfident may prompt them to change their mind and accept the alternative outcome (especially in computerized versions of the task that measure detection only based on choice “corrections,” e.g., Pärnamets et al., 2015, 2023; Strandberg et al., 2018). Similarly, reports of preference changes following exposure to false outcomes (e.g., Hall et al., 2013; Johansson et al., 2014; Strandberg et al., 2018, but see Taya et al., 2014; Rieznik et al., 2017) are likely to be traced back to changes of mind, combined with memory degradation.

### Limitations

Despite showing behavioral and pupillometry evidence of covert detection in two experiments, our study presents three important limitations. First, the number of available trials for some of the post-hoc comparisons of classified pupil responses reduced the reliability of the estimates and corresponding power. Hence, failure to detect differences in pupil responses between conditions may be due to the low power of the comparisons, especially when comparing unreported M-trials to (concurrently or retrospectively) reported M-trials and NM-trials.

Moreover, while we matched luminance, contrast, esthetic scores and semantic content (in Exp. 1) across image pairs, we cannot rule out the influence of additional features such as saliency and memorability. It is possible that if one image in a pair is highly salient or memorable, this could increase detection of manipulated responses, independent of the matched low-level and esthetic properties. Future studies could systematically investigate the contribution of these additional visual features to CB, for which we predict that manipulating salient and memorable choices would increase both concurrent and retrospective reporting rates.

Finally, as retrospective reports are only an indirect measure of detection and do not directly reflect concurrent (and sometimes concealed) detection, they are likely influenced by memory confounds, post-hoc retroactive inference and the memorability of the stimuli. Although the relationship between trial order and retrospective reports was weak in Exp. 1 and absent in Exp. 2, we observed that the sensitivity of retrospective reports was strongly modulated by the discriminability/memorability of the respective stimuli. This suggests that the reliability of retrospective reports is dependent on the experimental design. Previously reported low retrospective detection rates may therefore be partly explained by the use of rather forgettable stimuli, such as unfamiliar monochrome faces (e.g., Johansson et al., 2005).

### Restoring sight in choice blindness

Having established that many participants are not “choice blind” when presented with a false outcome, how can we make sense of the purported CB effect?

**Figure 5:**
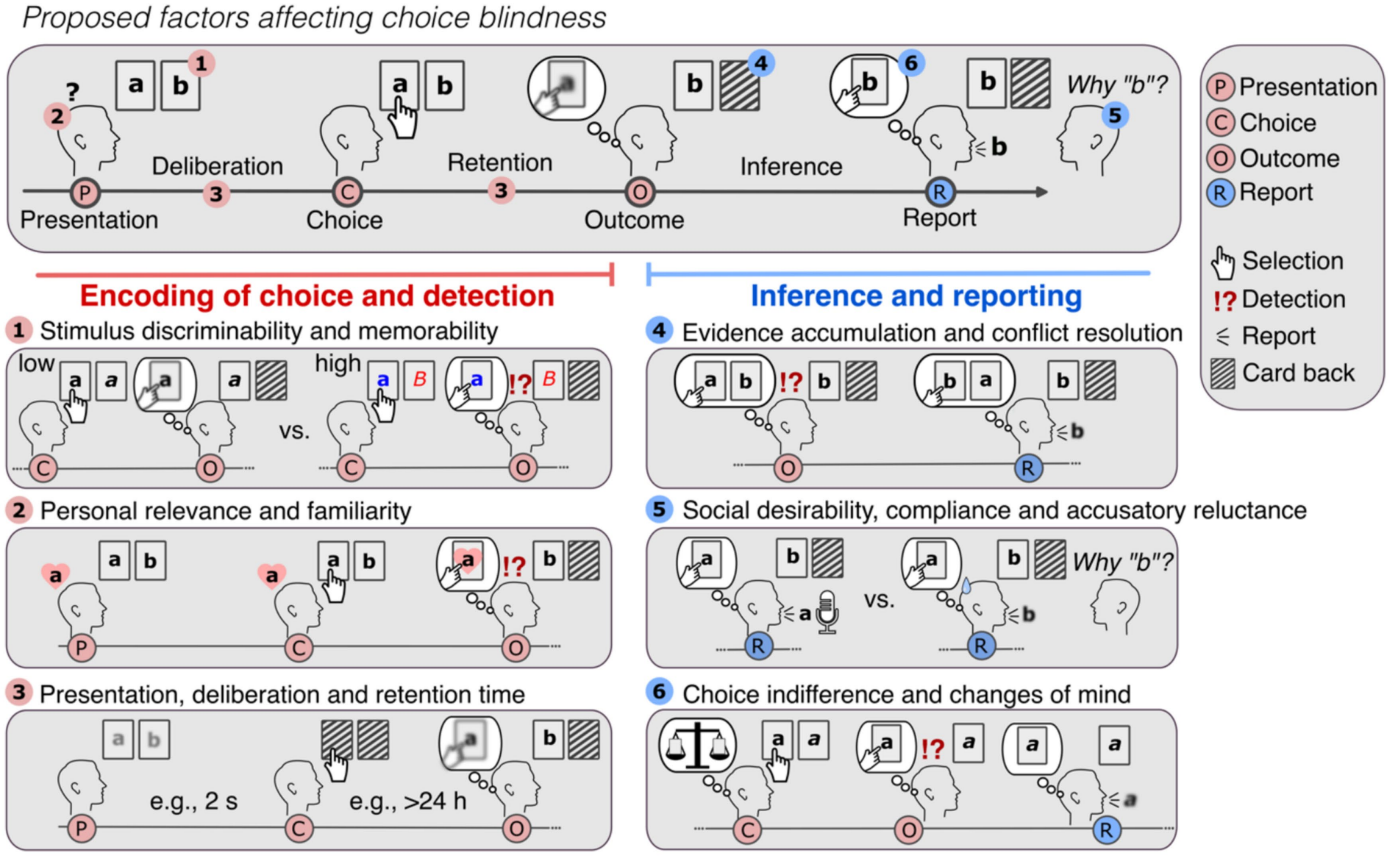
Potential factors affecting choice blindness. In a CB task, participants are generally presented with two options in card format (“a” and “b”) in the presentation and asked to decide between them. After their choice (e.g., “a”), and a (generally short) retention time, participants are presented with the non-selected option (e.g., “b”) (outcome) and asked to justify their choice (report). If participants have a weak or noisy representation of their choice (depicted as blurred representation), they may accept the new outcome and present reasons for it. Several factors may affect this effect. *Encoding of choice and detection*. As participants can only detect the manipulated outcome if they know and remember what their choice was, factors affecting the encoding of the choice will affect detection. Participants are likely to notice the false outcomes **(1)** if the stimuli are discriminable and memorable (e.g., as shown in Exp. 1 and 2), **(2)** if they are familiar and relevant for the participants (e.g., Somerville and McGowan, 2016) and finally, **(3)** if participants have enough time to decide (deliberation time) (Johansson et al., 2005) and are not required to retain their choice for too long to detect the false outcome (e.g., > 24 h as in Sauerland et al., 2013b; Sagana et al., 2014). *Inference and reporting*. **(4)** If participants know and remember their selection (“a”), they will notice when the alternative (“b”) is presented instead. Yet, if the experiments provide enough strong evidence for the altered choice, by, for example, using magic techniques to exchange the cards or showing the false outcome in the location of the original selection (as depicted), participants may be convinced that the manipulated outcome was their choice. Low reports rates in real-life CB experiments may be in part attributed to these deception methods, as holding on the original choice means either questioning the workings of the physical world or the integrity of the experiment. **(5)** In case participants detected the manipulation, social factors such as desirability, compliance and reluctance to accuse the researcher of tricking them, may negatively affect reporting. To avoid this bias toward no-reporting, experimental designs should aim to make reporting equally costly as not-reporting. **(6)** Finally, in case participants attribute the mismatch to honest errors (e.g., experiment malfunctioning, pressing the wrong button, etc.) and the altered choice is acceptable, participants might “change their mind” and simply accept the new outcome without signaling detection. Hence, while in each case participants may appear to be “choice blind,” in (4), they are in fact persuaded to believe otherwise (i.e., they update beliefs in view of strong evidence as rational agents do), in (5) they are reluctant to report and in (6) they knowingly accept the new altered outcome.

In CB tasks, participants are presented with their non-selected choice as their own. Participants may or may not detect this manipulation, and, provided they detect it, they may or may not report it. This separates CB tasks into two phases: the encoding and detection phase (consisting of stimulus presentation, choice and outcome) and the inference and reporting phase (see Figure 5).

In the first phase, the internal representation of the choice determines mismatch detection: if participants make choices they remember, the false outcome will necessarily come unexpected. Detection of false outcomes is thus likely when stimuli are discriminable and memorable (as shown in Exp. 2) and when they are familiar or of personal relevance (Sauerland et al., 2013b; Somerville and McGowan, 2016; Rieznik et al., 2017) (see Figure 5, left panel). In turn, if participants have only a weak or noisy representation of their choice, they are less likely to detect changes. Accordingly, detection is affected if presentation duration is brief (Johansson et al., 2005), if presentation of the manipulated outcomes occurs days after deciding (Sauerland et al., 2013b; Sagana et al., 2014), with increased demand and task difficulty (Sauerland et al., 2016), if participants are not paying attention (Petitmengin et al., 2013; Cheung et al., 2016) and if they forgot their original choice (Mouratidou, 2022).

In the second phase, if participants happen to miss the mismatch, they will provide ad-hoc reasons for the new outcome. In turn, if participants remember their selection, the second phase is characterized by inferential processes and conflict resolution (see Figure 5, right panel): the presentation of the unexpected outcome will violate their memory-based expectations and intentions. Participants will automatically look for the most likely solution for the unexpected outcome (e.g., “I pressed the wrong key,” “The experiment was malfunctioning”; as shown here), akin to how magic tricks elicit surprise and motivate viewers to explain them (Grassi and Bartels, 2021; Grassi et al., 2024). However, in experiments that use strong deception methods to alter the choices and reduce suspicion (such as magic techniques, e.g., Johansson et al., 2005; Hall et al., 2010, 2012, 2013), participants *may be persuaded* to believe that the manipulated outcome was their choice. They may then ascribe the mismatch to misremembering, as—in the common case—people take perceptual evidence at face value and trust others per default (Gilbert et al., 1990; Levine, 2014). Upon repeated unexpected outcomes or increased vigilance, participants may correctly identify the manipulations. Yet, as shown in our experiments, detection and reporting may diverge. Participants may withhold reporting because of false explanations, uncertainty, indifference, changes of mind (e.g., when the altered choice is acceptable) and social factors, such as social desirability, compliance, and “accusatory reluctance” (Ekman et al., 1980; O’Sullivan, 2003). We hypothesize that for participants to report the mismatches, the cost of having their choice altered needs to outweigh the cost of actively signaling distrust (cf. Ten Brinke et al., 2016) and response correction.

Hence, is it possible that—at least in some cases—instead of being “choice blind,” participants may make hasty, irrelevant decisions under circumstances that do not allow for a stable encoding of their choice (see Figure 5, points 1–3), be persuaded to question their memories/actions (4), be reluctant to signal distrust (5) and be indifferent about their choices (6).

## Conclusion

Overall, we present evidence that participants frequently detect but do not report the manipulated outcome in two computerized CB tasks. These covert detections may lead to the false impression that participants are “choice blind” and to an overestimation of the effect. Our results hence cast doubt on the general validity of CB, and with that on key conclusions of previous studies. Instead, our results suggest no failure of detection, but instead higher-level, cognitively or socially driven hesitance of reporting. CB is inherently limited (and often confounded) by a combination of perceptual, cognitive, motivational and social factors that affect detection (e.g., encoding of choice, stimulus discriminability, memorability, familiarity and personal relevance) and reporting (e.g., causal inference, cost of response correction, social desirability, changes of mind). Tracing back CB to these common-sense factors jointly provides a tentative interpretation that is more parsimonious and compatible with common-sense, folk-psychology and established accounts of decision-making, albeit less spectacular.

## Data Availability

The datasets presented in this study can be found in online repositories. The names of the repository/repositories and accession number(s) can be found below: Data and code can be found in: https://osf.io/q4ch6.
