# Supplementary material for: Restoring sight in choice blindness: pupillometry and behavioral evidence of covert detection
Source: Front Psychol. 2025 Dec 4;16:1598254. doi: 10.3389/fpsyg.2025.1598254 (PMC12713319; doi:10.3389/fpsyg.2025.1598254)
Supplement: Supplementary file 1 [file Data_Sheet_1.PDF]

Supplementary materials:

Restoring sight in choice blindness: pupillometry and behavioral evidence of covert detection

Pablo R. Grassi[1,2,3], Lena Hoeppe[4], Emre Baytimur[4,5], Andreas Bartels[1,2,3]
[1] Department of Psychology, University of Tübingen, Tübingen, Germany
[2] Centre for Integrative Neuroscience, Tübingen, Germany
[3] Max-Planck Institute for Biological Cybernetics, Tübingen, Germany
[4] Graduate Training Centre of Neuroscience, University of Tübingen, Tübingen, Germany
[5] Zurich Center for Neuroeconomics, Department of Economics, University of Zurich, Switzerland, current address

Sample size and statistical power for pupillometry analyses

Our experimental design in the first (Exp. 1) and second experiment (Exp. 2) had eight manipulated trials (M-trials) per participant to maximize power for pupillometry analyses, while maintaining a sample size comparable to previous CB studies (Johansson et al., 2008, 2014; Taya et al., 2014; Petitet et al., 2015). A similar CB experiment with eye-tracking had a total of 76 participants and an experimental design with eight M-trials (Pärnamets et al., 2023). For our power analysis, we used their openly available data to calculate the effect sizes (Cohens d) of pupil responses to M-trials (1.36), first reported M-trial (1.62), all reported trials M-trials (1.48), not reported M-trials (0.58) and between reported and not reported M-trials (0.72) (Dataset available in: https://osf.io/pf325/files/osfstorage). The number of participants necessary to detect such large effects of d > 1 in paired, one-sided comparisons at 80% power is n = 8 (alpha = 0.05; calculated using the "pwr" package in R, doi: 10.32614/CRAN.package.pwr). Detection of medium effects (d = 0.6) at 80% power can be achieved with n = 19 for one-sided or n = 24 for two-sided paired comparisons. Conversely, the minimum detectable effect sizes for paired comparisons at 80% power with the sample sizes of Exp. 1 (n = 35) are 0.43 (one-sided) and 0.48 (two-sided), and for Exp. 2 (n = 21), 0.56 (one-sided) and 0.64 (two-sided).

This suggests that both experiments should have adequate power to detect medium-to-large effects of pupil responses to concurrently reported M-trials (CR) and not concurrently reported M-trials (NCR). However, the exact sample sizes for each test depend on participants' reporting behavior and available eye tracking data (see Supplementary Tables S8-9). For example, while data from 35 participants was available in Exp. 1, only 27 had pupil data for concurrently reported trials. Moreover, not concurrently reported M-trials were further classified in "retrospective report" and "no report" trials (based on the retrospective memory task), which reduced trial counts in each condition and limited the reliability of the corresponding estimates. For example, only 11 participants in Exp. 1 had pupil data for "no report" M-trials. Therefore, failure to detect differences in pupil responses between post-hoc classified conditions with few paired samples may be due to the limited statistical power of some of these comparisons (n < 16 and n < 12 correspond to < 60% power for two- and one-sided tests, respectively).

Finally, to estimate the approximate power of our linear mixed-effects models, we simulated the ability to detect pupil responses to concurrently reported M-trials (large effect) and not concurrently reported M-trials (medium effect) based on the parameters estimates of a corresponding model fit to data from Pärnamets (2023)(using the "simr" package in R, Green and MacLeod, 2016) . We generated synthetic datasets in which each participant had 8 non-manipulated trials (NM-trials), 4 concurrently reported M-trials and 4 not concurrently reported M-trials (i.e., assuming a report rate of 50%). We modeled average pupil responses as a function of trial type,

including NM-trials, concurrently reported M-trials (CR), and not concurrently reported M-trials (NCR). Fixed-effects coefficients were set to 0.2127 for NM-trials (baseline), 0.6986 for CR M-trials, and 0.2359 for NCR M-trials. The model included a random intercept for each subject with variance of 0.145 and residual errors with variance of 0.432. We performed simulations for sample sizes ranging from 5 to 40 in steps of 5 to estimate the power to detect pupil responses in M-trials with and without concurrent report (1000 simulations per sample size, see Supplementary Figure S1). The simulations indicate that as few as 5 participants should be enough to detect pupil responses to reported M-trials with over 80% power (power 86.10%, CI-95%: [83.80, 88.19]). In contrast, more moderate pupil effects, such as those observed in not reported M-trials, required ca. 20 participants to achieve comparable power (power 83.90%, CI-95%: [81.47, 86.13]).

Supplementary Figures

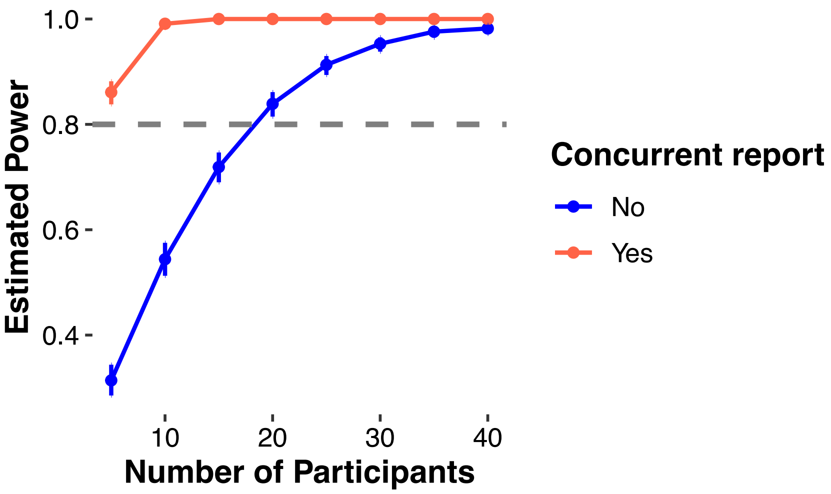

Figure S1. Power curves for simulated pupil responses. Estimated power for different sample sizes (range 5-40) is shown for detecting pupil responses to concurrently reported (red) and not concurrently reported (blue) M-trials in simulated pupil responses (1000 simulations per sample size). For concurrently reported M-trials, as few as five participants would achieve >80% power. Detection of the more modest effect observed in not concurrently reported M-trials requires at least 20 participants for comparable power. Error bars indicate 95% confidence intervals.

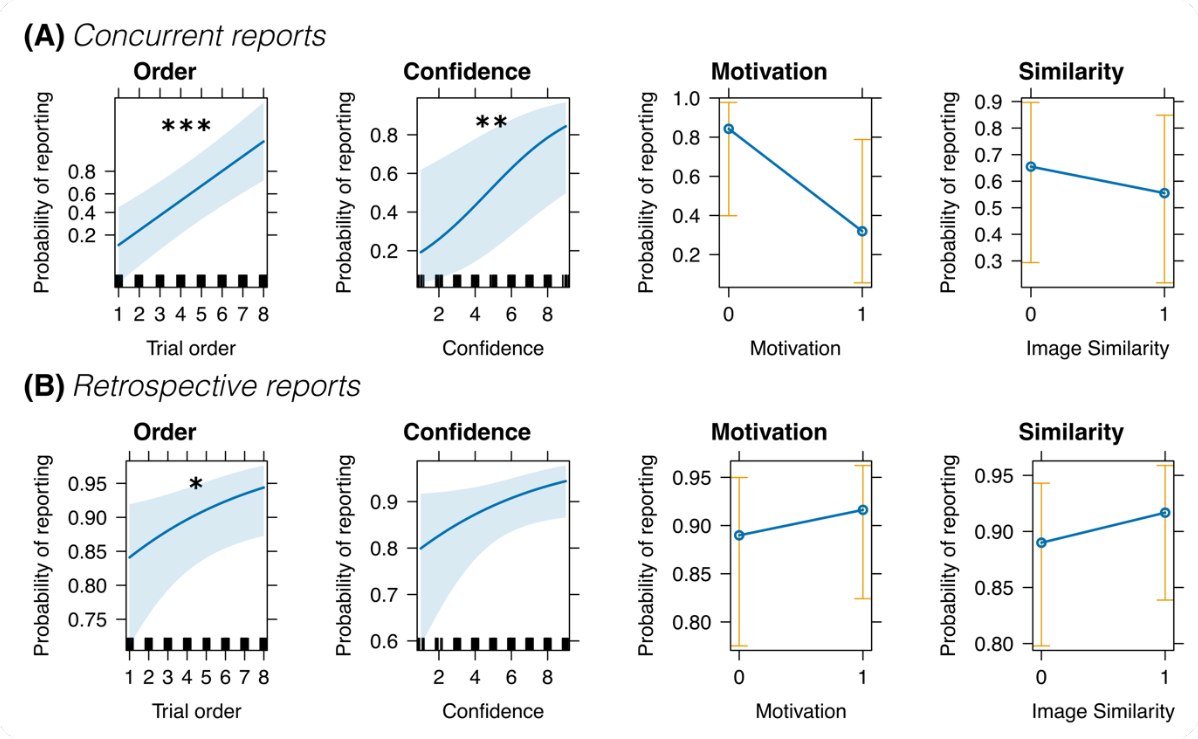

Figure S2. Experiment 1: logistic regression results. Shown are the predictors from the logistic regression for concurrent reports **(A)** and retrospective reports **(B)** from Exp. 1. Trial order and confidence in the responses significantly affected concurrent reporting rates but had less impact on retrospective reports. Against our expectations, motivation and image similarity did not affect reporting behavior. ***, $p < 0.001$; **, $p < 0.01$, *, $p < 0.05$.

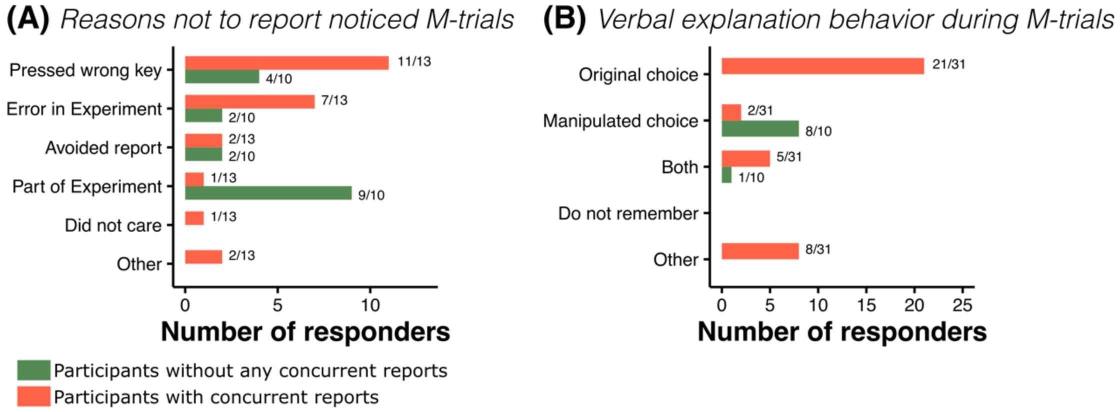

Figure S3. Experiment 1: post-experiment questionnaire responses. (A) Post-experiment questionnaire answers regarding reasons for not reporting M-trials, even when they were noticed, separated for participants with and without any concurrent report from Exp. 1. **(B)**. Post-experiment questionnaire answers about explanation behavior during M-trials in Exp. 1. As expected, most participants with concurrent reports claimed to have described their original choice when explaining their decision in M-trials. In contrast, most participants without any concurrent report (8 out of 10), stated that they had reported the manipulated choice instead. Hence, without a memory-based detection task, many trials from these participants would have been classified as “no detection”, even though they had actually noticed the changes.

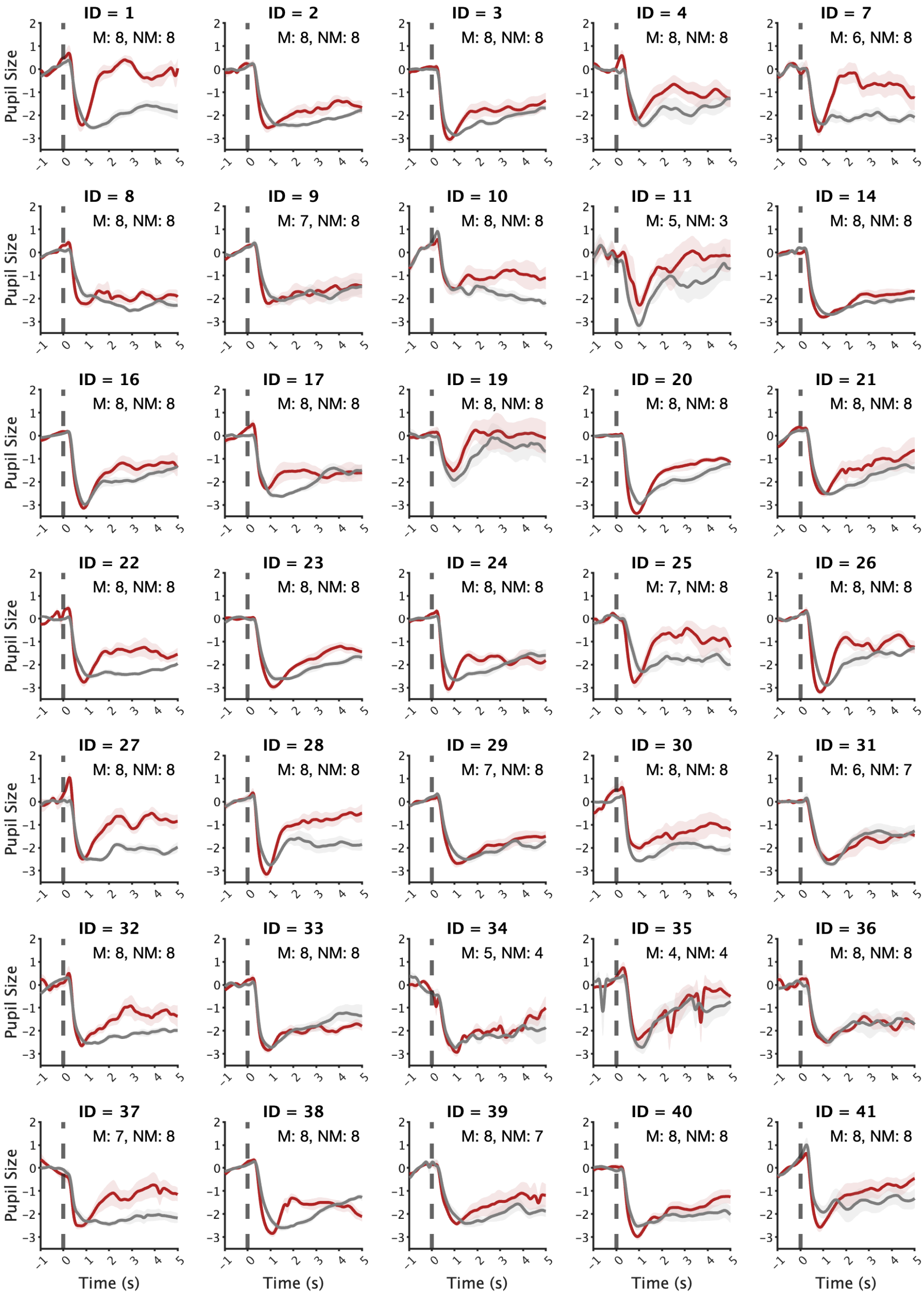

Figure S4. Experiment 1: single subject pupillometry data. Shown are the mean pupil traces (± SEM) for manipulated (M, red) and non-manipulated (NM, grey) trials of all subjects from Exp. 1 included in the pupillometry analyses for visualization (N = 35). The number of M-trials and NM-trials per participant is indicated in each panel. In accord to the group analysis, pupil responses in M-trials are larger compared to NM-trials in most participants.

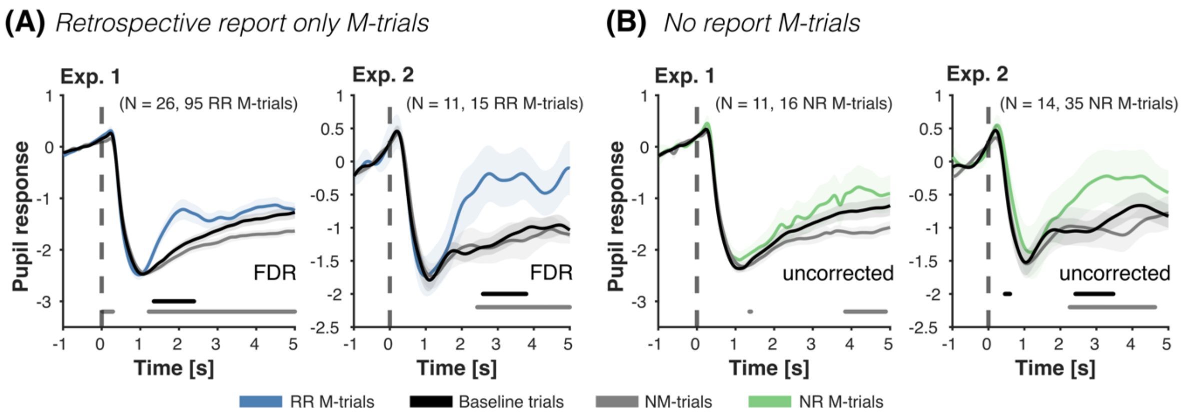

Figure S5. Pupil responses during retrospective report only and no-report M-trials. Shown are mean pupil size time series (± SEM) for retrospective report only manipulated trials (RR M-trials) **(A)** and no-report M-trials (NR M-trials) **(B)** for Exp. 1 and Exp. 2 along with non-manipulated (NM-trials, grey) and baseline trials (black) for visualization. Horizontal lines parallel to x-axis show significant differences (one-sided paired t-tests, FDR corrected or uncorrected) to NM-trials (grey line) and baseline trials (black line). In NR M-trials, no time point survived FDR-correction. Note that the reliability of the estimates is limited by the small number of available trials in some of the conditions.

Supplementary Tables

Supplementary Table S1. Experiment 1: responses to final question. Shown are responses to the final question asked before debriefing to see if participants (n = 41) noticed something special about the experiment (Exp. 1). Please note that seven participants closed the experiment without providing an answer, but all reported to have noticed the manipulations to the experimenter. Only one participant showed no indication of detection before debriefing (i.e., no concurrent report and no indication of detection during the final question). Please note that some of the responses have been shortened for readability and translated from German. Responses without any indication of detection are shown in **bold** (n = 4). n.a.: no answer.

SUBJECT	RESPONSES TO FINAL QUESTION (BEFORE DEBRIEFING)	ADDITIONAL NOTES
**S01**	n.a.	Participant reported to have noticed manipulations.
**S02**	“Um, some of the pictures were, um, incorrectly assigned, so to speak. Or in other words, that I selected the picture although I selected the other one.”	
**S03**	n.a.	Participant reported to have noticed manipulations.
**S04**	“Sometimes I select a picture, but I am then shown the other picture. So, what I choose and what I mean are two different things. […]”	
**S05**	“Um, yes, so images were also selected from time to time, or it was suggested to you that you had selected them although you had actually chosen the other motif. The first time, I was still unsure whether I had simply clicked the wrong one, but then it happened more often and sometimes with images that had a strong – that is, where it was already relatively clear which one was most likely rated as the better one by the majority. Sometimes, however, with motifs that were almost the same, or that showed the same motif but just from a different perspective, I did briefly question whether I had chosen the wrong one after all... […]”.	
**S06**	n.a.	Participant reported to have noticed manipulations.
**S07**	“I noticed that whenever I said I was confident that the image that I selected was chosen by many people it always showed me the other image and not the one that I selected.”	
**S08**	“Yes, certain times when I am about to choose an image the software seems to automatically choose an image for me. And that really is not my choice in most cases. […]”	
**S09**	**“No, I didn't really notice anything special. […]”**	
**S10**	“Some of the pictures I selected with the keyboard were not the ones that were displayed.”	
**S11**	“[…] and I noticed that very often a different picture was displayed than the one I clicked on. […]”	
**S12**	“Yes, that the images I chose were not the ones I was asked why I chose them.”	
**S13**	“Yes, there were some images that when I pressed them, they were asking me the opinion about the other one basically. […]”	
**S14**	**“No”**	
**S15**	“I had a bit of a problem with the experiment in that the right-left button didn't work - that wasn't intentional. […]”	
**S16**	“I mean apart from sometimes not showing the right picture... I mean it would not show the picture that I selected [...]”	
**S17**	“I was often shown images that I had not selected.”	
**S18**	n.a.	Participant reported to have noticed manipulations.
**S19**	“There were times that the answer that I [had] chosen did not pop on the screen. For the first two times, I did not mention that the image that I [had] chosen was not presented but I went to explain why I found the other one impressive […].”	
**S20**	“Well, sometimes the other image is shown than the one I select?”	
**S21**	“Yes, I think sometimes – I don't know in which percentage but often –, I was asked about the reason of my choice but asking why I chose the things that I didn't actually choose. […]”	
**S22**	“Yeah, sometimes I noticed about the experiment that sometimes the images [were] swapped when I was asked why I selected a certain image.”	
**S23**	“Yes, in some trials, the image that I perceived as more beautiful was not displayed, but the other one.”	
**S24**	“So sometimes the pictures I didn't choose came up, I don't know if that was intentional or not. […]”	
**S25**	“Well, yes, but I thought that it was a mistake of the system that I chose one picture and then I saw the other and then I had to explain about the other picture. […]”	
**S26**	n.a.	Participant reported to have noticed the manipulations.
**S27**	“[…] I noticed that sometimes the pictures I selected were not displayed […].”	
**S28**	n.a.	Participant reported to have noticed the manipulations.

**S29**	“Some of the pictures that I choose are not shown at the end, so I am not sure if I pressed the arrow wrongly or it is on purpose.”	
**S30**	“Yes, at half of the images they put me, they told me that I picked the other one. So… yeah, I still think that… I still stick to my choice. […]”	
**S31**	**“Something special I noticed about the experiment is how the quality of some pictures are quite different in terms that some pictures have very high quality and other ones have much lower quality.”**	
**S32**	“The selection wasn't always right, which I chose to explain, so to speak. It was perhaps 50:50.”	
**S33**	“I noticed that images are repeatedly displayed that I have not selected.”	
**S34**	“During the experiment, the image that I selected did not appear. Sometimes, the image that I didn't [select appear], and I [had] to explain why that image was more aesthetic.”	
**S35**	“Yeah, I do notice that sometimes it would show me the other image rather than the one that I chose. And I am pretty sure I missed one wrong choice. That means, like, I chose the correct image I thought but then it showed the other one and I gave the explanation. And I am pretty sure I did it only once. […]”	
**S36**	n.a.	Participant reported to have noticed the manipulations.
**S37**	“I noticed that it didn't really matter which button I pressed, because sometimes one image was displayed as if I had selected it and sometimes the other, regardless of which button I pressed. Sometimes the controls were practically inverted, or a picture was randomly displayed, and I was told that I had selected it.”	
**S38**	**“I didn't notice anything special.”**	
**S39**	“Sometimes I wasn't sure if I had pressed the right button. And some of the pictures were very similar in terms of aesthetics and quality.”	
**S40**	“That the answers that were displayed were sometimes incorrect.”	
**S41**	“From time to time I was shown the wrong picture that I had not selected.”	

Supplementary Table S2. Experiment 1: example responses of subjects with full concurrent reports. Listed are verbal responses to all M-trials (M1-8) for four example subjects that concurrently reported all M-trials from Exp. 1. As depicted below, all participants already provided tentative explanations to the detected M-trials (“I clicked wrong”, “there is something wrong with the code”, etc.). Please note that some of the responses have been shortened for readability and translated from German. The first concurrent report (CR) of each participant with a tentative explanation is shown in **bold**. M1-8: M-trial number; CR: concurrent report; RR: retrospective report.

SUBJECT	TRIAL	VERBAL RESPONSES	CR	RR
**S05**	**M1**	**“So, either I clicked wrong, or … I actually chose the other image, because the other one […]”**	**Yes**	**No**
	**M2**	“So, I actually selected the other image again. So, either I clicked wrong or it's part of the experiment. I chose the other picture […].”	**Yes**	**Yes**
	**M3**	“So, I actually chose the other motif. For the reason that it had more colors, […].”	**Yes**	**Yes**
	**M4**	“I actually chose the other image again. Because that one also had […]”	**Yes**	**Yes**
	**M5**	“I think I chose for the other image. But I didn't find either image so convincing, I […].”	**Yes**	**Yes**
	**M6**	“I chose the other picture and not this one, because the other one showed […].”	**Yes**	**Yes**
	**M7**	“I actually chose for the other picture because it somehow came across as […].”	**Yes**	**Yes**
	**M8**	“I chose the other picture and I'm pretty sure that the majority probably chose the […].”	**Yes**	**Yes**
**S08**	**M1**	**“This was not the image I selected. I used the right arrow, but this came up... […] This was not the image that I chose. […] So, this was not the image I chose, but somehow the software is showing this image.”**	**Yes**	**Yes**
	**M2**	“No. I haven't selected this image. I chose the left one. So, I think there is a mistake in the software.”	**Yes**	**No**
	**M3**	“I did not select this image. I chose the other one. There is a mistake in the software.”	**Yes**	**Yes**
	**M4**	“I did not choose this image. I chose the other one. There is some mistake in the software.”	**Yes**	**No**
	**M5**	“I did not choose this image. There is some mistake in the software. I chose the other.”	**Yes**	**Yes**
	**M6**	“[…] Continuously it is giving me the wrong image to explain. [...] I did not choose this image I chose the other one. There is a mistake I think in the software.”	**Yes**	**No**
	**M7**	“I did not choose this image.”	**Yes**	**Yes**
	**M8**	“I did not choose this image.”	**Yes**	**No**
**S16**	**M1**	“Sorry, but I thought I selected the other image. But …um… the other image […].”	**Yes**	**Yes**
	**M2**	**“[…] Are you here? I don't think it is registering my right button correctly. Because two times when I pressed it, it said that I selected the left image when I selected the right one. […]”**	**Yes**	**No**
	**M3**	“Again, I think I selected the other image because this one looks a bit dull and […].”	**Yes**	**Yes**
	**M4**	“So, I have selected the other image, cause it had nicer colors and nicer […].”	**Yes**	**Yes**
	**M5**	“Again, I selected the other image cause it had nicer colors. This one seems dull, […].”	**Yes**	**Yes**
	**M6**	“Again, I selected the other image cause this one has duller colors and there is a […].”	**Yes**	**Yes**
	**M7**	“I selected the other one again because it was more symmetrical, and I like […].”	**Yes**	**Yes**
	**M8**	“So again, I selected the other image. I like the composition of the other image […].”	**Yes**	**Yes**
**S28**	**M1**	“I actually thought I chose the other picture, but ok. I personally think it's more […]”	**Yes**	**No**
	**M2**	**“I didn't choose this picture. I chose the other one, um and either there's something wrong with the code or I don't know but I'm pretty sure I chose the other one. So, I'll continue now.”**	**Yes**	**Yes**
	**M3**	“I chose the other picture, and I really don't like it. I don't actually find either of them particularly beautiful […]”	**Yes**	**Yes**
	**M4**	“I didn't choose this picture. I chose the other one because I think it was too […].”	**Yes**	**No**
	**M5**	“Did I really choose this one, yes?”	**Yes**	**No**
	**M6**	“I chose the other one. The other one had warmer colors, and that's why I think the other one appealed to more people.”	**Yes**	**Yes**
	**M7**	“I didn't choose this picture. I thought the other one was much nicer. It's somehow too […].”	**Yes**	**Yes**
	**M8**	“As I did not select this, there is no explanation now.”	**Yes**	**No**

Supplementary Table S3. Experiment 1: example responses of subjects with some concurrent reports. Listed are all verbal responses to all M-trials (M1-8) for four example subjects with some concurrently reported M-trials from Exp. 1. Importantly, each of these participants also show indications of covert detection. Please note that some of the responses have been shortened for readability and translated from German. The first concurrent report (CR) of each participant is shown in **bold**. M1-8: M-trial number; CR: concurrent report; RR: retrospective report.

SUBJECT	TRIAL	VERBALS RESPONSES	CR	RR
**S01**	**M1**	"Because the background is more diverse".	**No**	**Yes**
	**M2**	"Because of the autumnal red tones."	**No**	**Yes**
	**M3**	"Because of the blue color, which goes well with the sea."	**No**	**Yes**
	**M4**	"Because of the more varied wings."	**No**	**Yes**
	**M5**	"Because the house looks special."	**No**	**Yes**
	**M6**	**"Just a quick question. I'm pretty sure I selected the other image. Sometimes I thought I had chosen the other one and wasn't sure if I was wrong, but this time I'm very sure […]"**	**Yes**	**Yes**
	**M7**	"[…] Ok, I chose the other picture."	**Yes**	**Yes**
	**M8**	"Because the contrasts come out better here."	**No**	**Yes**
**S07**	**M1**	"I like the colors in the image and […] the other image is not very colorful, so I think […]."	**No**	**Yes**
	**M2**	**"I actually selected the other image, I think. Have I misplaced the keys? I think I selected the other image, have I misplaced the keys? […] Ok, because it happened like twice. This […]."**	**Yes**	**Yes**
	**M3**	"This is not the image I selected […]. Ok, this is not the image that I selected. But the other image was where the photo was not edited so it looked more natural and […]."	**Yes**	**Yes**
	**M4**	"Hm, this image has a lot of colors, and it shows the contrast between the sky and […]."	**No**	**Yes**
	**M5**	"I did not select this image. I chose the other one. And I like that image better […]."	**Yes**	**Yes**
	**M6**	"I have not selected this image. I chose the other image. I think that the other image […]."	**Yes**	**Yes**
	**M7**	"This is not the image that I selected. I selected the other image. And I feel that the […]."	**Yes**	**Yes**
	**M8**	"I chose the other image, and I chose the other image because it is more clear. This […]."	**Yes**	**Yes**
**S33**	**M1**	"The colors were stronger."	**No**	**Yes**
	**M2**	**"I have a question. I'm pretty sure that this is the second time I've seen a picture that I didn't select. […] Ok, so I didn't say anything the first time but... this is not the picture I chose […]"**	**Yes**	**Yes**
	**M3**	"This is not the picture I chose. The other one I chose had stronger colors and […]."	**Yes**	**Yes**
	**M4**	"This is not the picture I chose. The other picture at night somehow had the nicer coloring."	**Yes**	**Yes**
	**M5**	"This is not the picture I chose. The symmetry of the other picture somehow made it […]."	**Yes**	**Yes**
	**M6**	"I chose the other picture because it somehow looked better with the fog."	**Yes**	**Yes**
	**M7**	"This is not the picture I chose, because the other one […]."	**Yes**	**Yes**
	**M8**	"I chose the other picture because it has a better point of view […]"	**Yes**	**Yes**
**S38**	**M1**	"It looks more natural."	**No**	**Yes**
	**M2**	**"Are you still in the room? It has happened twice now that I selected a different picture. […]"**	**Yes**	**Yes**
	**M3**	"I chose the other image because this image also looks very ordinary and the other […]."	**Yes**	**Yes**
	**M4**	"I chose the other image because it looked more posed and here it is, with the red […]."	**Yes**	**Yes**
	**M5**	"I chose the other image. I think the place here is more beautiful than the […]."	**Yes**	**Yes**
	**M6**	"I chose the other image. Here is the […]."	**Yes**	**Yes**
	**M7**	"I chose the other image. I think […]."	**Yes**	**Yes**
	**M8**	"I chose the other image. The water looked more unnatural, not so real."	**Yes**	**Yes**

Supplementary Table S4. Experiment 1: example responses of subjects with no concurrent reports. Listed are verbal responses to all M-trials (M1-8) for four example subjects without any concurrent report from Exp. 1. All participants without any concurrent report (n = 10), retrospectively correctly identified at least three M-trials. Responses to the final question (FQ) before debriefing indicate covert detection and are shown in **bold**. Please note that some of the responses have been shortened for readability and translated from German. M1-8: M-trial number; FQ: final question; CR: concurrent report; RR: retrospective report.

SUBJECT	TRIAL	VERBALS RESPONSES	CR	RR
**S17**	**M1**	"It is very detailed, and the background is beautifully blurred."	**No**	**Yes**
	**M2**	"The picture has great colors."	**No**	**Yes**
	**M3**	"The water plays around the stones and is very detailed."	**No**	**Yes**
	**M4**	"The pattern is very nice, and the colors and the clouds still shine through the object."	**No**	**Yes**
	**M5**	"The picture has a dynamic quality."	**No**	**Yes**
	**M6**	"The colors of the sky and the meadow are very rich in contrast and the sky [...]."	**No**	**Yes**
	**M7**	"The red of the house is very rich in contrast to the rest of the picture and […]."	**No**	**Yes**
	**M8**	"The transition between sharp and blurred objects, meaning, between stones and […]."	**No**	**Yes**
	**FQ**	**"I was often shown images that I had not selected."**		
**S34**	**M1**	"I think this image is more attractive than the other one, because it depicted […]."	**No**	**Yes**
	**M2**	"I think this image is more attractive than the other one, because of the contrast, […]."	**No**	**Yes**
	**M3**	"I find this image more attractive than the other one, because of the use of […]."	**No**	**Yes**
	**M4**	"I think this image is more aesthetic than the other one, because it depicted […]."	**No**	**Yes**
	**M5**	"I find this image more aesthetic than the other one, because this image used […]."	**No**	**Yes**
	**M6**	"I find this image more aesthetic than the other one, because this image has […]."	**No**	**Yes**
	**M7**	"I find this image more aesthetic than the other one, because it captures the […]."	**No**	**Yes**
	**M8**	"I think this image is more aesthetic than the other one, because it […]."	**No**	**Yes**
	**FQ**	**"During the experiment, the image that I selected did not appear. Sometimes, the image that I didn't [select appear], and I [had] to explain why that image was more aesthetic."**		
**S40**	**M1**	"Because it is good weather."	**No**	**Yes**
	**M2**	"Because of the sunset."	**No**	**Yes**
	**M3**	"Because of the colors."	**No**	**Yes**
	**M4**	"Because of the different colors."	**No**	**Yes**
	**M5**	"Because the dog looks around and you don't know what's there."	**No**	**Yes**
	**M6**	"Because the colors of the tree and the grasses match."	**No**	**Yes**
	**M7**	"Because there is more river on it."	**No**	**Yes**
	**M8**	"Because the tiger doesn't look so scary."	**No**	**Yes**
	**FQ**	**"That the answers that were displayed were sometimes incorrect."**		
**S41**	**M1**	"Beautiful color combination, autumn-like."	**No**	**No**
	**M2**	"Warm colors, high in detail."	**No**	**Yes**
	**M3**	"House by the sea, very high in contrast."	**No**	**Yes**
	**M4**	"Natural"	**No**	**Yes**
	**M5**	"High in contrast, beautiful tree, detailed moss."	**No**	**Yes**
	**M6**	"Beautiful sunset, light coming through the clouds."	**No**	**No**
	**M7**	"Beautiful hiking area, sharp."	**No**	**Yes**
	**M8**	"More natural."	**No**	**Yes**
	**FQ**	**"From time to time, I was shown the wrong picture that I had not selected."**		

Supplementary Table S5. Experiment 2: example responses with low and high confidence. Listed are selected verbal responses from M-trials of five subjects in Exp. 2, illustrating the difference between low- and high-confidence choices. Low-confidence choices often occurred when images had similar attractiveness ratings, and participants did not concurrently report manipulations. Instead, they expressed indifference and/or a "change of mind". Please note that some of the responses have been shortened for readability and translated from German. Low confidence answers are shown in **bold**, high-confidence answers in *italic*. M1-8: M-trial number; Sim.: similar rating; Conf.: reported confidence (from 1 to 9); CR: concurrent report; RR: retrospective report.

SUBJECT	TRIAL	VERBAL RESPONSES	SIM.	CONF.	CR	RR
**S09**	**M1**	*"Oh... um, actually I chose the other one, I thought.... but ok... [Laughs] but she is attractive too ... so I guess... I wasn't ... that sure... ok."*	**Yes**	**5**	**Yes**	**Yes**
	**M2**	**"Um, I think her face looks very symmetrical to me. So, I think that made ... her more attractive."**	**No**	**4**	**No**	**No**
**S13**	**M2**	*"Um, […] I don't think I selected them at all ... I think I pressed left. Can that be? […]"*	**No**	**7**	**Yes**	**No**
	**M3**	**"Um, I couldn't decide this time who I found more attractive and chose ... chose one of the two people at random."**	**Yes**	**3**	**No**	**Yes**
**S18**	**M2**	**"I have a preference for […]. But ... both were very similar, so I am not too confident about my decision."**	**No**	**2**	**No**	**No**
	**M5**	*"Um, I misclicked. I wanted the other one and I prefer […] I wanted to say the other one."*	**No**	**7**	**Yes**	**No**
**S19**	**M1**	**"Um, I actually thought I chose the other person. But, um, I did not really find any of two more or less attractive. I thought the other one had […] I thought I preferred that. So, I think, I actually eventually chose this one."**	**Yes**	**1**	**Yes**	**No**
	**M2**	*"Wait... what? ... Ok, maybe I am stupid today, but I definitely chose the other person […] I thought the other person was a lot prettier than the one that I am currently shown, because […]. So, the other person was more attractive."*	**No**	**9**	**Yes**	**Yes**
**S22**	**M1**	*"Huh? I have ... May I have a moment? [...] I think I mistyped. I typed left, and now the right image is displayed [...]. Well, I actually chose the left-hand picture [...]."*	**No**	**8**	**Yes**	**Yes**
	**M4**	**"Gut feeling"**	**Yes**	**4**	**No**	**Yes**

Supplementary Table S6. Experiment 1: comparison of averaged pupil data between conditions. Shown are t-tests of manipulated (M-) and non-manipulated (NM-) trials against baseline trials and between the different M-trials (concurrently reported, CR; not concurrently reported, NCR; retrospectively reported, RR and no report, NR). Note that NCR contain both RR and NR M-trials and that the DFs change because of differences in reporting behavior between participants (CR, NCR, RR and NR). $^+$, right-tailed tests. P-values < 0.05 are in **bold**.

T-TEST	T	DF	P-VALUE	MEAN	95%-CI	COHENS D
**NM**	-2.55	34	**0.015**	-0.19	[-0.35, -0.04]	-0.43
**M$^+$**	3.74	34	**<0.001**	0.37	[0.21, Inf]	0.63
**CR$^+$**	3.87	26	**<0.001**	0.48	[0.27, Inf]	0.74
**NCR$^+$**	2.21	27	**0.02**	0.36	[0.08, Inf]	0.42
**RR$^+$**	1.86	25	**0.037**	0.31	[0.026, Inf]	0.36
**NR$^+$**	0.66	10	0.26	0.24	[-0.41, Inf]	0.20
**CR-NM$^+$**	6.25	26	**<0.001**	0.67	[0.49, Inf]	1.2
**NCR-NM$^+$**	4.23	27	**<0.001**	0.56	[0.34, Inf]	0.8
**RR-NM$^+$**	4.02	25	**<0.001**	0.53	[0.30, Inf]	0.79
**NR-NM$^+$**	1.54	10	0.08	0.5	[-0.09, Inf]	0.46
**NCR-CR**	-0.11	19	0.9	-0.02	[-0.45, 0.41]	-0.02
**NR-CR**	-0.61	6	0.57	-0.33	[-1.63, 0.98]	-0.23
**NR-RR**	-0.09	8	0.93	-0.03	[-0.79, 0.73]	-0.03
**CR-RR**	0.21	17	0.83	0.04	[-0.44, 0.41]	0.05

Supplementary Table S7. Experiment 2: comparison of averaged pupil data between conditions. Shown are t-tests of manipulated (M-) and non-manipulated (NM-) trials against baseline trials and between the different M-trials (concurrently reported, CR; not concurrently reported, NCR; retrospectively reported, RR and no report, NR). Note that NCR contain both RR and NR M-trials and that the DFs change because of differences in reporting behavior between participants (CR, NCR, RR and NR). $^+$, right-tailed tests. P-values < 0.05 are in **bold**.

T-TEST	T	DF	P-VALUE	MEAN	95%-CI	COHENS D
**NM**	-0.64	20	0.531	-0.09	[-0.39, 0.21]	-0.14
**M$^+$**	3.17	20	**0.002**	0.51	[0.23, Inf]	0.69
**CR$^+$**	2.49	19	**0.01**	0.43	[0.13, Inf]	0.55
**NCR$^+$**	3.16	15	**0.003**	0.75	[0.33, Inf]	0.79
**RR$^+$**	3.02	10	**0.006**	0.9	[0.36, Inf]	0.91
**NR$^+$**	1.99	13	**0.03**	0.57	[0.06, Inf]	0.53
**CR-NM$^+$**	2.38	19	**0.01**	0.47	[0.13, Inf]	0.53
**NCR-NM$^+$**	3.35	15	**0.002**	0.73	[0.35, Inf]	0.84
**RR-NM$^+$**	2.93	10	**0.007**	0.94	[0.36, Inf]	0.88
**NR-NM$^+$**	2.53	13	**0.01**	0.66	[0.2, Inf]	0.67
**NCR-CR**	1.07	14	0.3	0.23	[-0.24, 0.70]	0.28
**NR-CR**	0.43	12	0.7	0.11	[-0.44, 0.66]	0.12
**NR-RR**	-2.98	8	**0.02**	-0.86	[-1.53, -0.2]	-0.99
**CR-RR**	-1.95	9	0.08	-0.61	[-1.31, 0.1]	-0.62

Supplementary Table S8. Experiment 1: pupillometry trial counts. Shown are the number of valid manipulation (M), no manipulation (NM) and baseline trials used in the pupillometry analysis. A trial was considered invalid if more than 2 s of data had to be interpolated. Participants were not included in the pupillometry analysis if more than 15 trials were invalid (S05, S06, S13, S15, S18) and no data available data (S12). The total number of participants included in the pupillometry analyses was N = 35.

SUBJECT	N_M	N_{NM}	$N_{BASELINE}$	$N_{INVALID}$	INCLUDED
**S01**	8	8	14	0	Yes
**S02**	8	8	14	0	Yes
**S03**	8	8	14	0	Yes
**S04**	8	8	13	1	Yes
**S05**	**0**	**5**	**7**	**18**	**No**
**S06**	**0**	**0**	**0**	**30**	**No**
**S07**	6	8	14	2	Yes
**S08**	8	8	14	0	Yes
**S09**	2	8	13	2	Yes
**S10**	8	8	14	0	Yes
**S11**	5	3	9	13	Yes
**S12**	**-**	**-**	**-**	**-**	**No data**
**S13**	**0**	**0**	**1**	**29**	**No**
**S14**	8	8	14	0	Yes
**S15**	**1**	**1**	**4**	**24**	**No**
**S16**	8	8	14	0	Yes
**S17**	8	8	14	0	Yes
**S18**	**1**	**1**	**0**	**28**	**No**
**S19**	8	8	12	2	Yes
**S20**	8	8	14	0	Yes
**S21**	8	8	14	0	Yes
**S22**	8	8	14	0	Yes
**S23**	8	8	14	0	Yes
**S24**	8	8	14	0	Yes
**S25**	7	8	12	3	Yes
**S26**	8	8	14	0	Yes
**S27**	8	8	14	0	Yes
**S28**	8	8	14	0	Yes
**S29**	7	8	10	5	Yes
**S30**	8	8	14	0	Yes
**S31**	6	7	14	3	Yes
**S32**	8	8	14	0	Yes
**S33**	8	8	14	0	Yes
**S34**	5	4	9	12	Yes
**S35**	4	4	14	8	Yes
**S36**	8	8	14	0	Yes
**S37**	7	8	14	1	Yes
**S38**	8	8	14	0	Yes
**S39**	8	7	13	2	Yes
**S40**	8	8	14	0	Yes
**S41**	8	8	14	0	Yes

Supplementary Table S9. Experiment 2: pupillometry trial counts. Shown are the number of valid manipulation (M), no manipulation (NM) and baseline trials used in the pupillometry analysis. A trial was considered invalid if more than 2 s of data had to be interpolated. Participants were not included in the pupillometry analysis if more than 15 trials were invalid (S06) or had no available data (S23). The total number of participants included in the pupillometry analyses was N = 21.

SUBJECT	N_{M}	N_{NM}	$N_{BASELINE}$	$N_{INVALID}$	INCLUDED
**S01**	8	7	13	2	Yes
**S02**	6	6	11	7	Yes
**S03**	7	7	11	5	Yes
**S04**	8	7	12	3	Yes
**S05**	8	8	14	0	Yes
**S06**	**2**	**5**	**5**	**18**	**No**
**S07**	8	8	13	1	Yes
**S08**	2	7	9	12	Yes
**S09**	3	7	12	8	Yes
**S10**	7	7	13	3	Yes
**S11**	8	8	14	0	Yes
**S12**	8	7	12	3	Yes
**S13**	8	8	14	0	Yes
**S14**	8	8	14	0	Yes
**S15**	6	7	12	5	Yes
**S16**	8	8	14	0	Yes
**S17**	3	5	12	10	Yes
**S18**	8	8	14	0	Yes
**S19**	5	8	14	3	Yes
**S20**	7	6	9	8	Yes
**S21**	7	7	9	7	Yes
**S22**	4	7	9	10	Yes
**S23**	**-**	**-**	**-**	**-**	**No data**

References

Green, P., and MacLeod, C. J. (2016). SIMR : an R package for power analysis of generalized linear mixed models by simulation. *Methods Ecol Evol* 7, 493–498. doi: 10.1111/2041-210X.12504

Johansson, P., Hall, L., and Sikström, S. (2008). FROM CHANGE BLINDNESS TO CHOICE BLINDNESS. *An International Journal of Psychological Sciences* 51, 142–155. doi: 10.2117/psysoc.2008.142

Johansson, P., Hall, L., Tärning, B., Sikström, S., and Chater, N. (2014). Choice Blindness and Preference Change: You Will Like This Paper Better If You (Believe You) Chose to Read It!: Choice Blindness and Preference Change. *J. Behav. Dec. Making* 27, 281–289. doi: 10.1002/bdm.1807

Pärnamets, P., Johansson, P., Strandberg, T., Balkenius, C., and Hall, L. (2023). Looking at choice blindness: Evidence from gaze patterns and pupil dilation. doi: 10.31234/osf.io/v85sz

Petitet, P., Noonan, M. A. P., Bridge, H., O'Reilly, J. X., and O'Shea, J. (2015). Testing the inter-hemispheric competition account of visual extinction with combined TMS/fMRI. *Neuropsychologia* 74, 63–73. doi: 10.1016/j.neuropsychologia.2015.04.021

Taya, F., Gupta, S., Farber, I., and Mullette-Gillman, O. A. (2014). Manipulation Detection and Preference Alterations in a Choice Blindness Paradigm. *PLoS ONE* 9, e108515. doi: 10.1371/journal.pone.0108515
